# Supplementary material for: Discovery of 2-(1-(3-(4-Chloroxyphenyl)-3-oxo- propyl)pyrrolidine-3-yl)-1H-benzo[d]imidazole-4-carboxamide: A Potent Poly(ADP-ribose) Polymerase (PARP) Inhibitor for Treatment of Cancer
Source: Molecules. 2019 May 17;24(10):1901. doi: 10.3390/molecules24101901 (PMC6572064; doi:10.3390/molecules24101901)
Supplement: Supplementary file 1 [file molecules-24-01901-s001.zip › HRMS of 5ca-5cp.pdf]

136

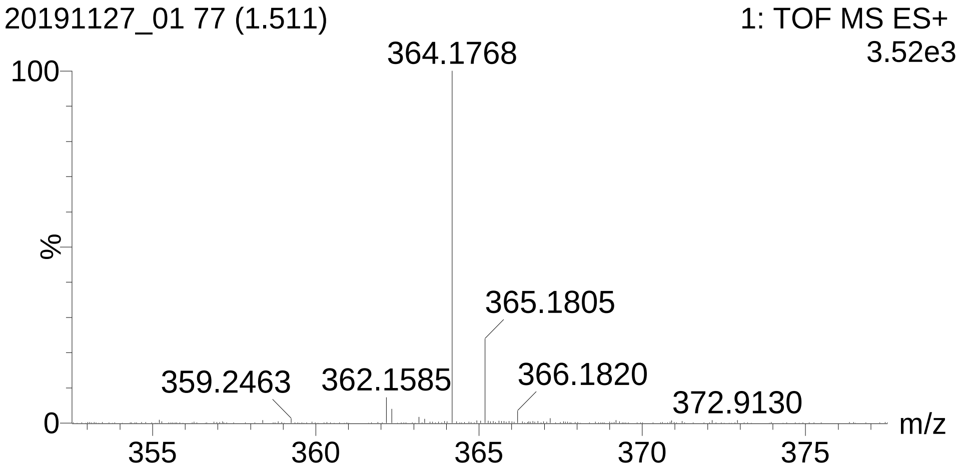

20191127_01 77 (1.511)
1: TOF MS ES+
3.52e3
364.1768
365.1805
359.2463
362.1585
366.1820
372.9130
100
%
0
m/z
355
360
365
370
375

137

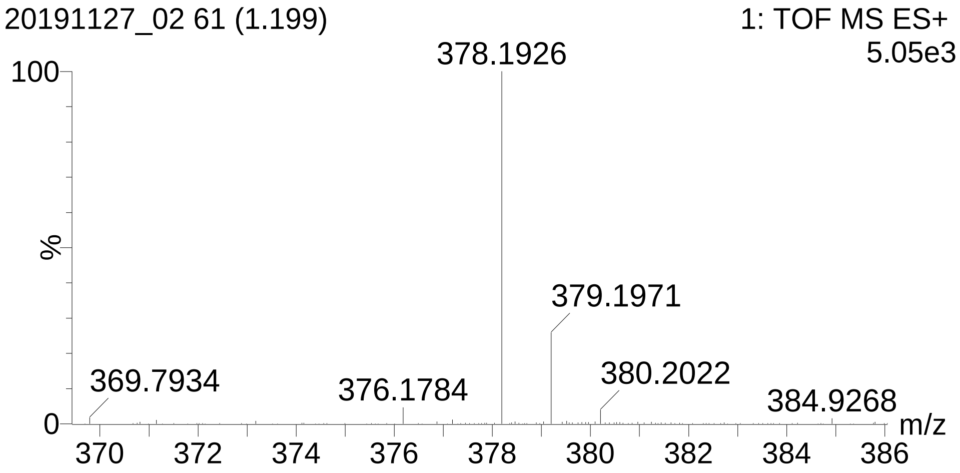

20191127_02 61 (1.199)
1: TOF MS ES+
5.05e3
378.1926
379.1971
369.7934
376.1784
380.2022
384.9268
100
%
0
m/z
370
372
374
376
378
380
382
384
386

138

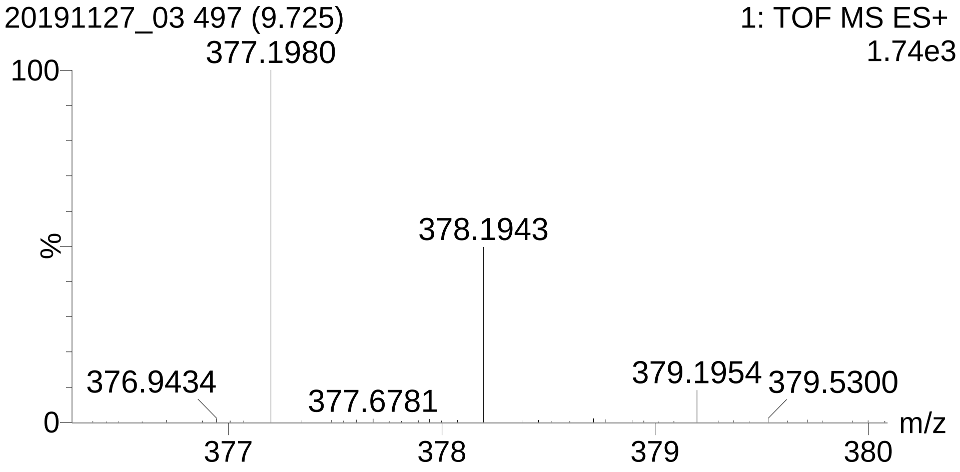

20191127_03 497 (9.725)
1: TOF MS ES+
1.74e3
377.1980
378.1943
376.9434
377.6781
379.1954
379.5300
100
%
0
m/z
377
378
379
380

142

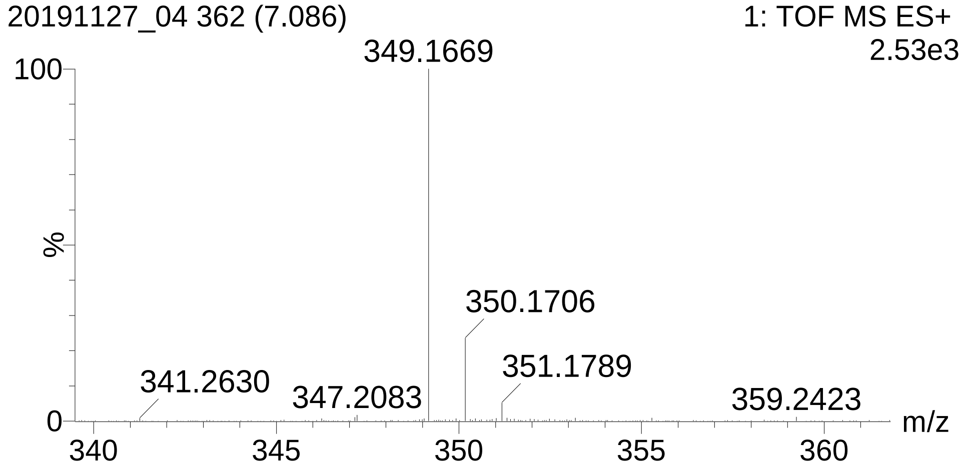
20191127_04 362 (7.086)
1: TOF MS ES+
2.53e3
349.1669
350.1706
351.1789
341.2630
347.2083
359.2423
100
%
0
340
345
350
355
360
m/z

143

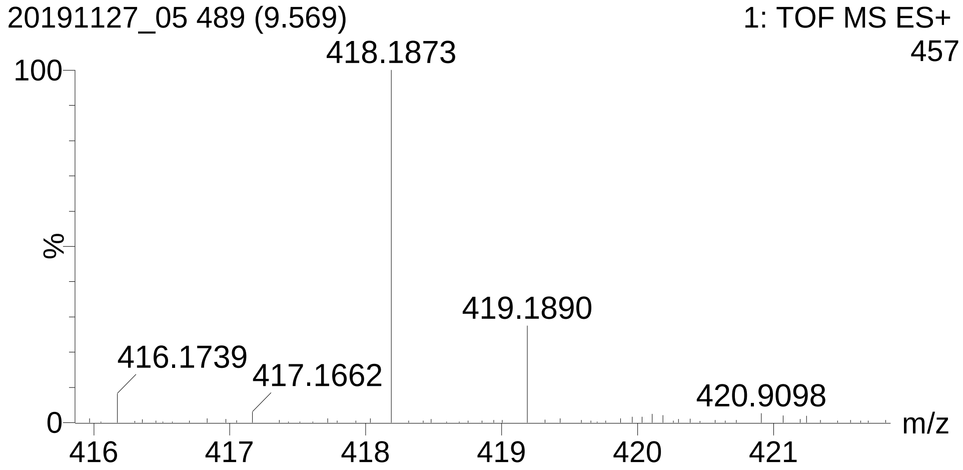
20191127_05 489 (9.569)
1: TOF MS ES+
457
418.1873
419.1890
416.1739
417.1662
420.9098
100
%
0
416
417
418
419
420
421
m/z

146

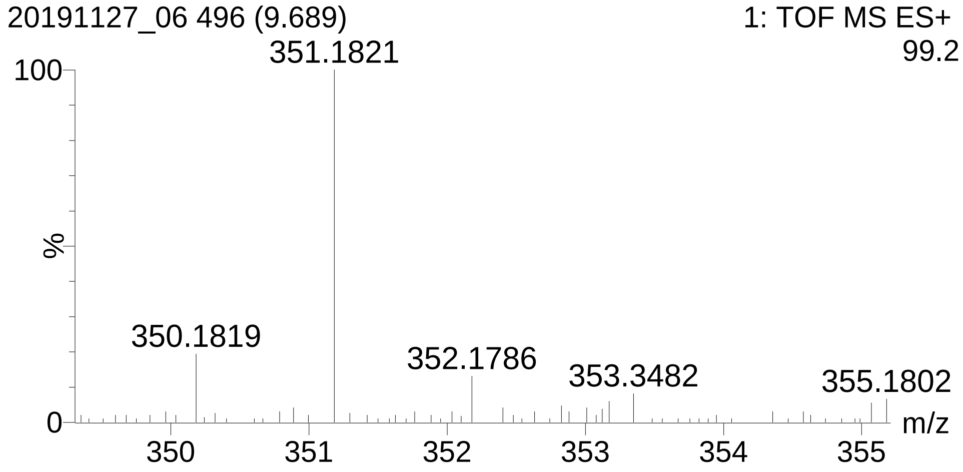
20191127_06 496 (9.689)
1: TOF MS ES+
99.2
351.1821
350.1819
352.1786
353.3482
355.1802
100
%
0
350
351
352
353
354
355
m/z

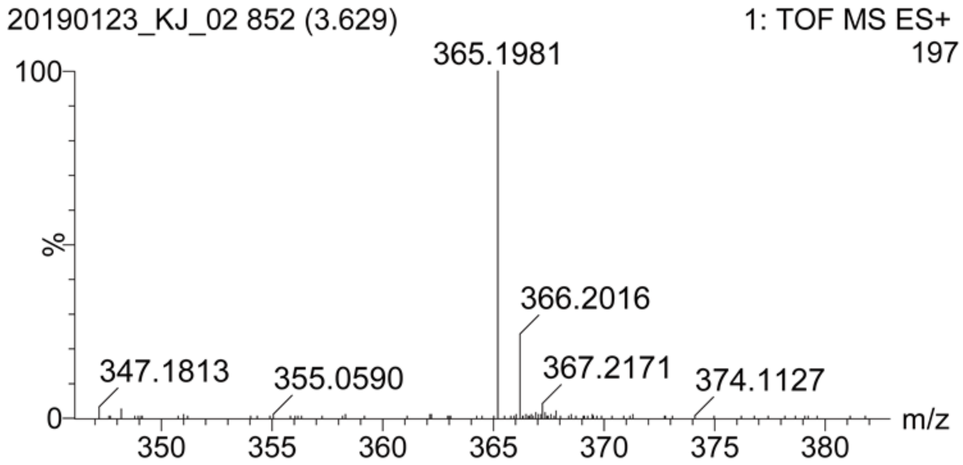
20190123_KJ_02 852 (3.629)
1: TOF MS ES+
197
365.1981
100
%
0
366.2016
347.1813
355.0590
367.2171
374.1127
m/z
350
355
360
365
370
375
380

157

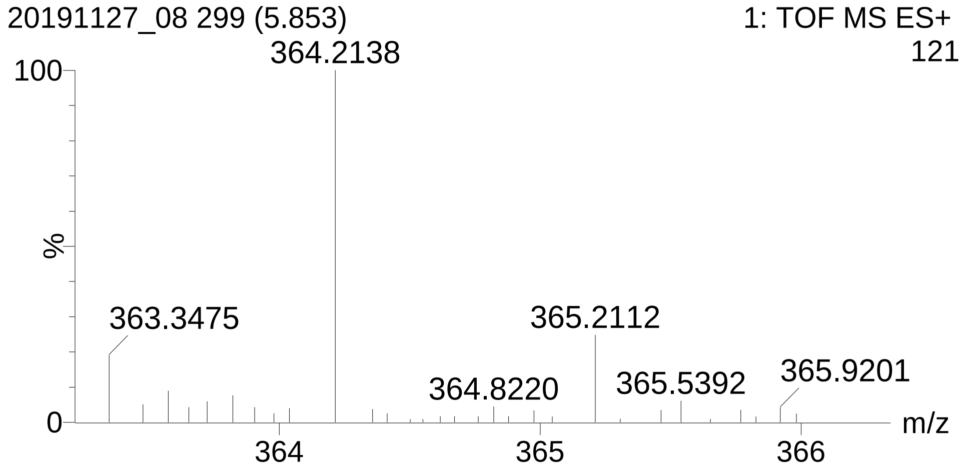
20191127_08 299 (5.853)
1: TOF MS ES+
121
364.2138
100
%
0
363.3475
365.2112
364.8220
365.5392
365.9201
m/z
364
365
366

171

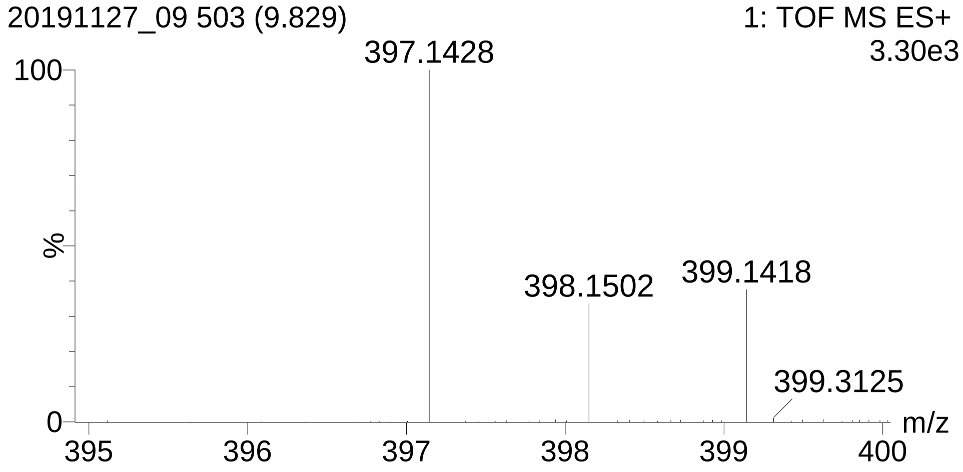
20191127_09 503 (9.829)
1: TOF MS ES+
3.30e3
397.1428
100
%
0
398.1502
399.1418
399.3125
m/z
395
396
397
398
399
400

172

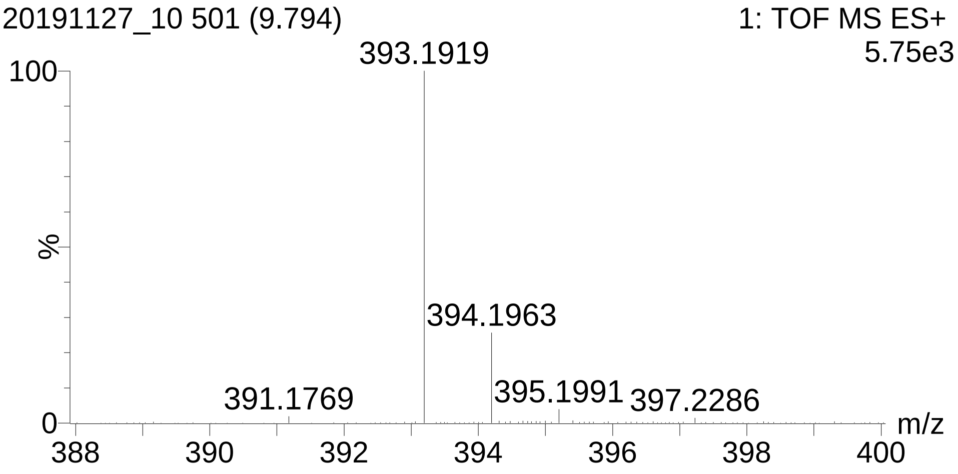
20191127_10 501 (9.794)
1: TOF MS ES+
5.75e3
393.1919
394.1963
391.1769
395.1991
397.2286
100
%
0
m/z
388
390
392
394
396
398
400

15

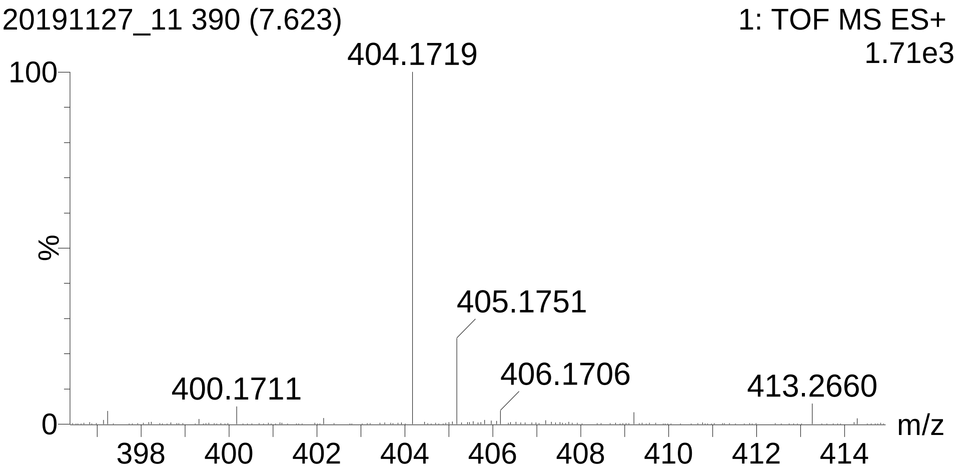
20191127_11 390 (7.623)
1: TOF MS ES+
1.71e3
404.1719
405.1751
400.1711
406.1706
413.2660
100
%
0
m/z
398
400
402
404
406
408
410
412
414

22

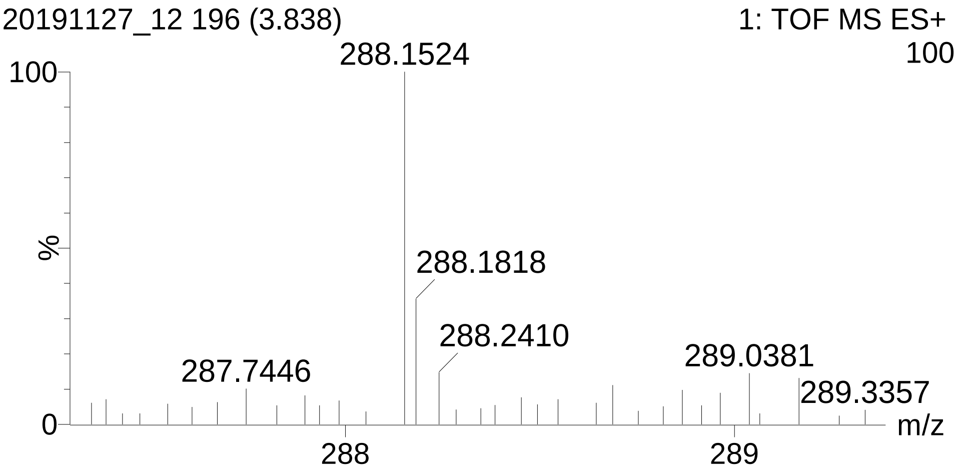
20191127_12 196 (3.838)
1: TOF MS ES+
100
288.1524
288.1818
288.2410
287.7446
289.0381
289.3357
100
%
0
m/z
288
289

27

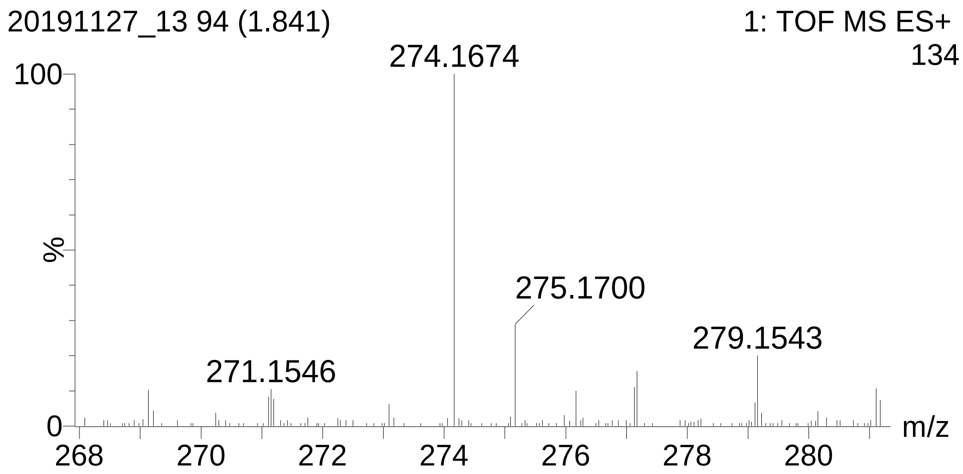
20191127_13 94 (1.841)
1: TOF MS ES+
134
274.1674
275.1700
279.1543
271.1546
100
%
0
m/z
268
270
272
274
276
278
280

30

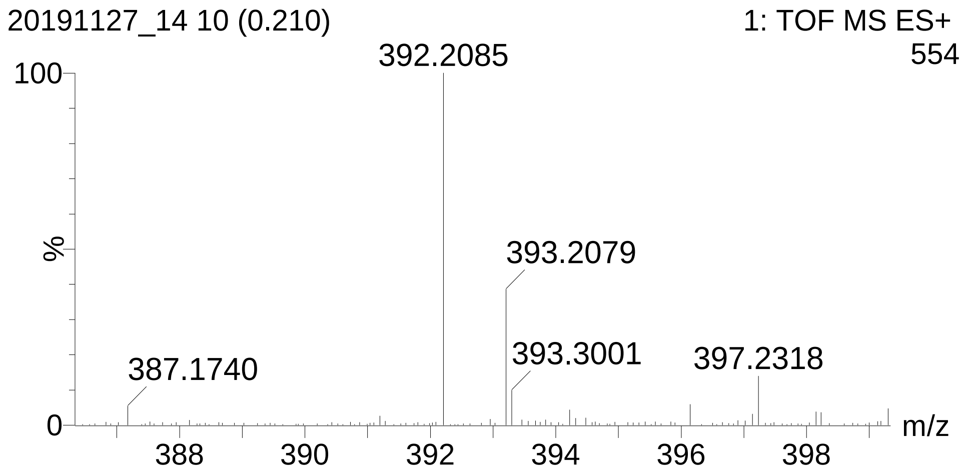
20191127_14 10 (0.210)
1: TOF MS ES+
554
392.2085
393.2079
393.3001
397.2318
387.1740
100
%
0
m/z
388
390
392
394
396
398

31

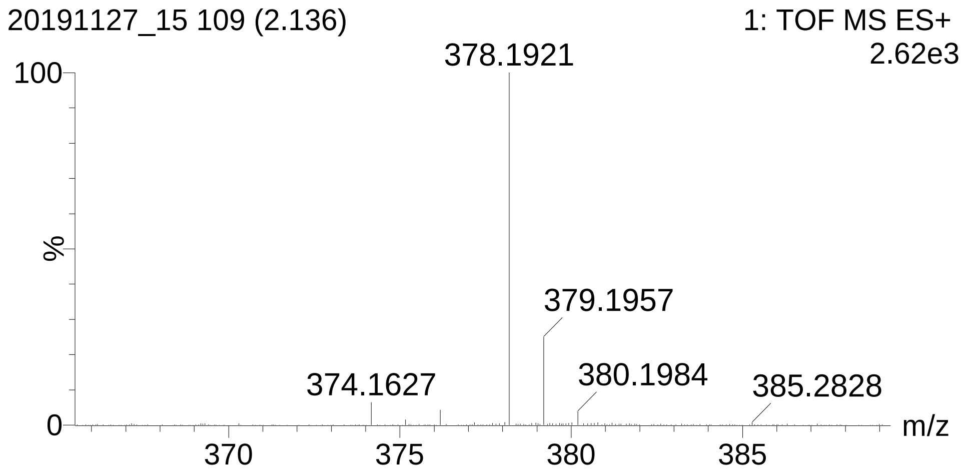
20191127_15 109 (2.136)
1: TOF MS ES+
2.62e3
378.1921
379.1957
380.1984
385.2828
374.1627
100
%
0
m/z
370
375
380
385

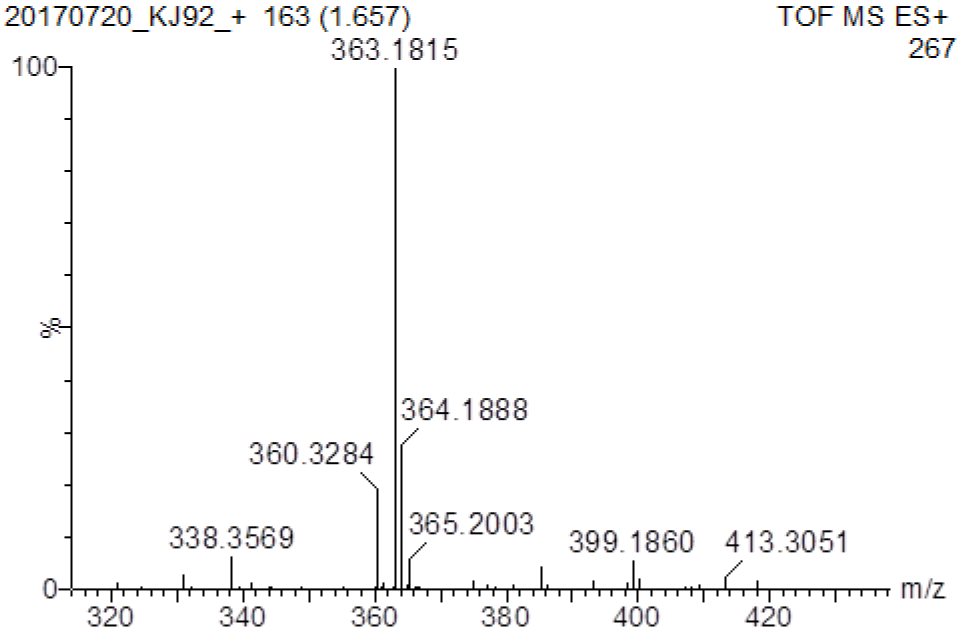

20170720_KJ92_+ 163 (1.657)
TOF MS ES+
267
363.1815
100
%
0
364.1888
360.3284
365.2003
338.3569
399.1860
413.3051
320
340
360
380
400
420
m/z
