# Supplementary material for: Discovery of 2-(1-(3-(4-Chloroxyphenyl)-3-oxo- propyl)pyrrolidine-3-yl)-1H-benzo[d]imidazole-4-carboxamide: A Potent Poly(ADP-ribose) Polymerase (PARP) Inhibitor for Treatment of Cancer
Source: Molecules. 2019 May 17;24(10):1901. doi: 10.3390/molecules24101901 (PMC6572064; doi:10.3390/molecules24101901)
Supplement: Supplementary file 1 [file molecules-24-01901-s001.zip › NMR of 5ca-5cp.pdf]

5ca

^{1}H NMR (400 MHz, Methanol-d_4) δ 7.86 (d, J = 7.6 Hz, 1H), 7.67 (d, J = 8.0 Hz, 1H), 7.54 – 7.49 (m, 2H), 7.31 – 7.24 (m, 3H), 7.11 – 7.05 (m, 1H), 3.85 – 3.76 (m, 1H), 3.52 – 3.39 (m, 2H), 3.17 (dd, J = 6.7, 2.4 Hz, 2H), 3.11 – 3.03 (m, 1H), 2.93 – 2.84 (m, 1H), 2.53 – 2.41 (m, 1H), 2.34 (ddd, J = 12.9, 8.4, 6.5 Hz, 1H).

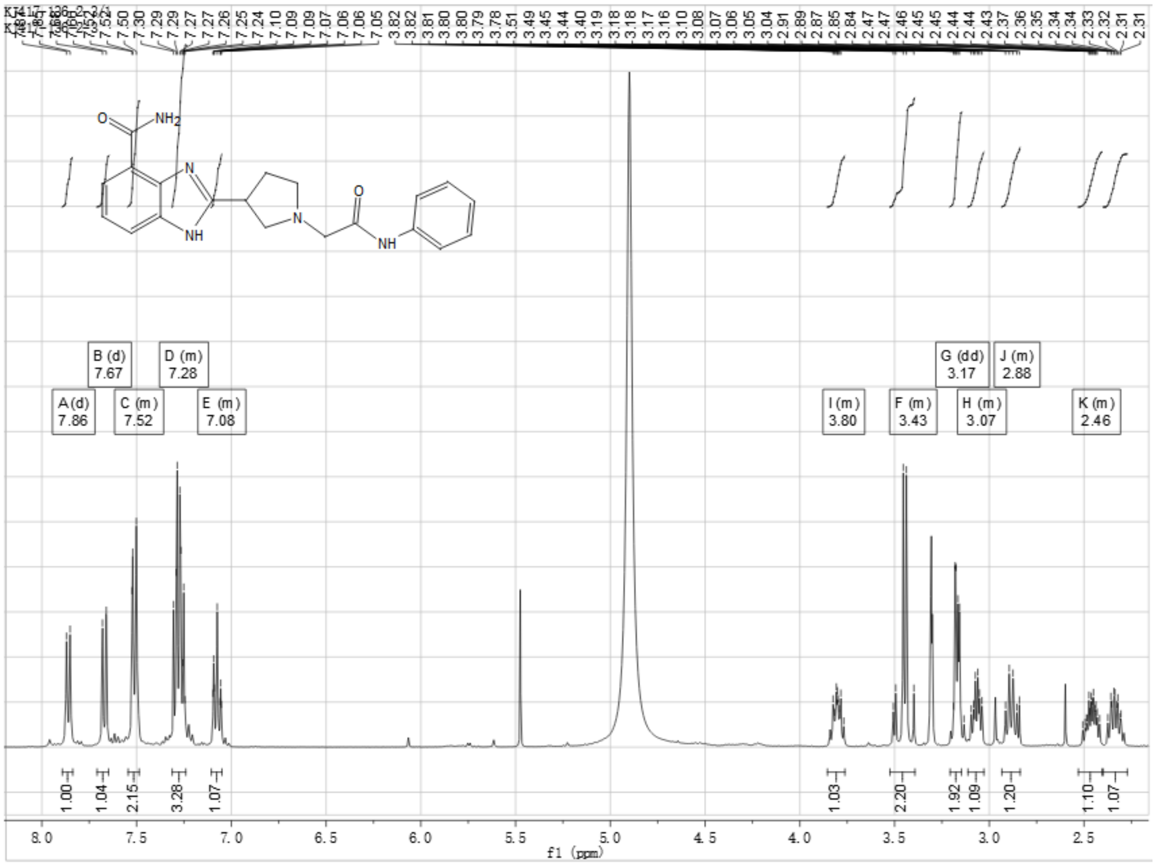

^{13}C NMR (101 MHz, MeOD) δ 169.26, 158.72, 137.71, 128.44, 124.09, 122.23, 121.48, 119.98, 58.66, 58.29, 53.45, 37.37, 30.02.

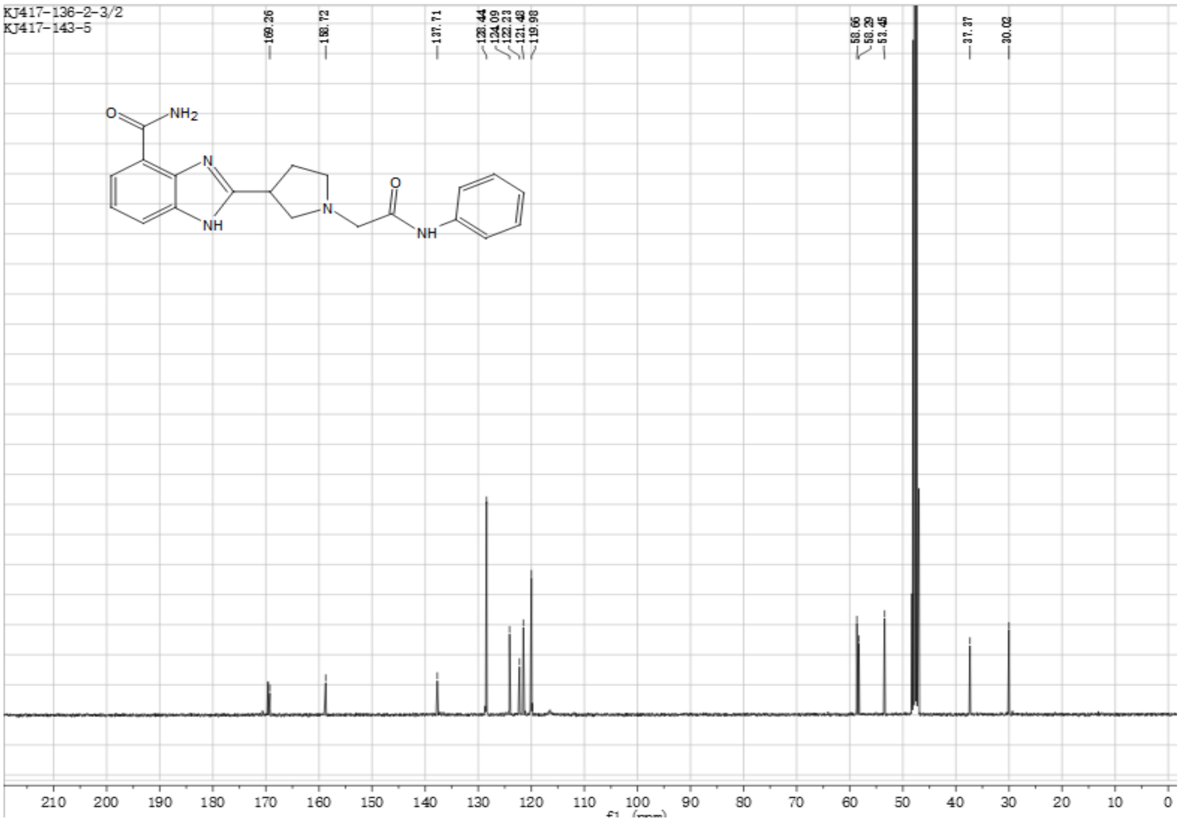

5cb (containing acetone signal)

^{1}H NMR (400 MHz, Methanol-d_4) δ 7.86 (d, J = 7.6 Hz, 1H), 7.63 (d, J = 8.0 Hz, 1H), 7.53 – 7.40 (m, 2H), 7.35 – 7.16 (m, 3H), 7.10 – 6.97 (m, 1H), 3.79 (dt, J = 13.1, 6.3 Hz, 1H), 3.20 (d, J = 7.4 Hz, 2H), 3.11 – 2.96 (m, 3H), 2.96 – 2.84 (m, 1H), 2.67 (t, J = 6.9 Hz, 2H), 2.53 – 2.28 (m, 2H).

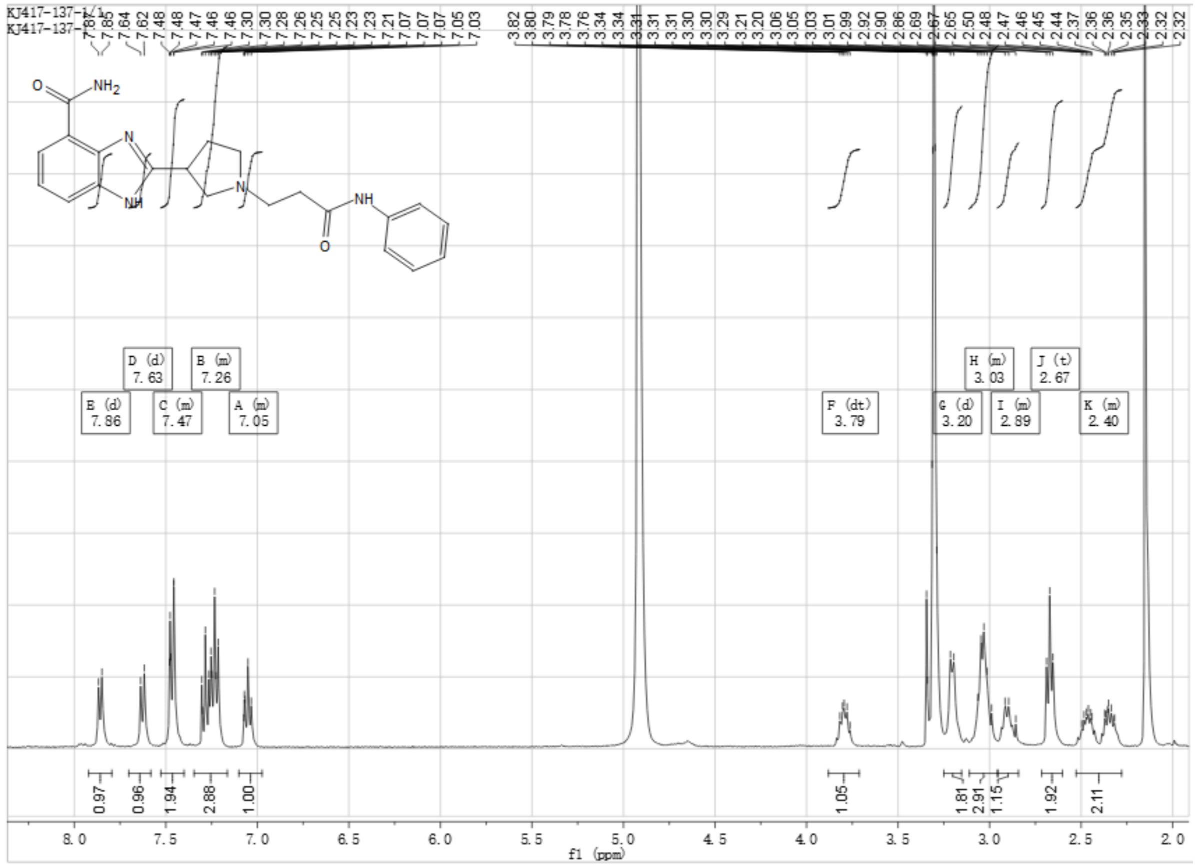

^{13}C NMR (101 MHz, MeOD) δ 171.18, 169.20, 158.26, 138.29, 128.37, 123.79, 122.27, 121.48, 119.80, 58.22, 53.03, 51.21, 37.01, 34.86, 29.55.

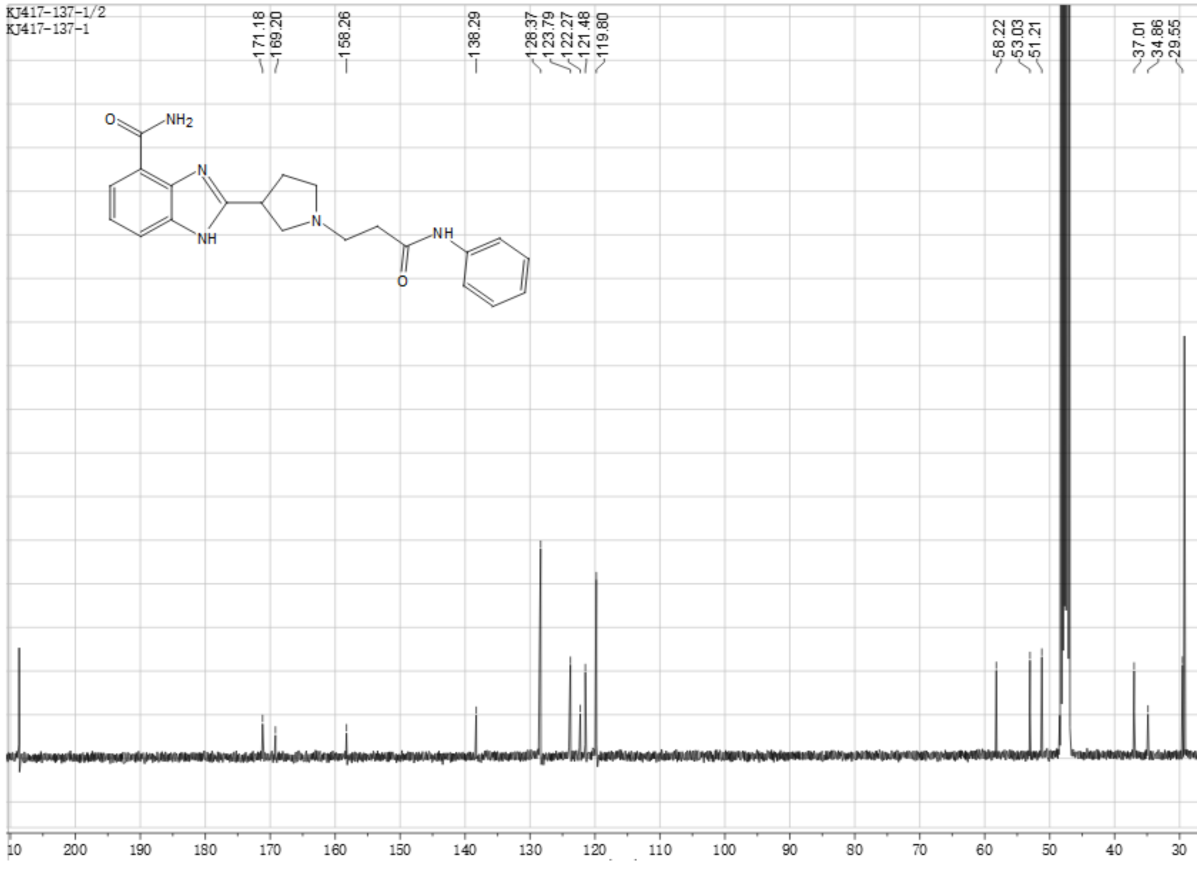

5cc (containing acetone signal)

^{1}H NMR (400 MHz, Methanol-d_4) δ 8.07 – 7.97 (m, 2H), 7.88 (d, J = 7.7 Hz, 1H), 7.69 (d, J = 8.0 Hz, 1H), 7.65 – 7.57 (m, 1H), 7.50 (dd, J = 7.6 Hz, 2H), 7.32 (dd, J = 8.9, 6.7 Hz, 1H), 3.81 – 3.65 (m, 1H), 3.16 (dd, J = 11.1, 7.8 Hz, 2H), 3.04 – 2.77 (m, 4H), 2.74 – 2.61 (m, 2H), 2.50 – 2.37 (m, 1H), 2.35 – 2.23 (m, 1H), 2.02 (dd, J = 7.2 Hz, 2H).

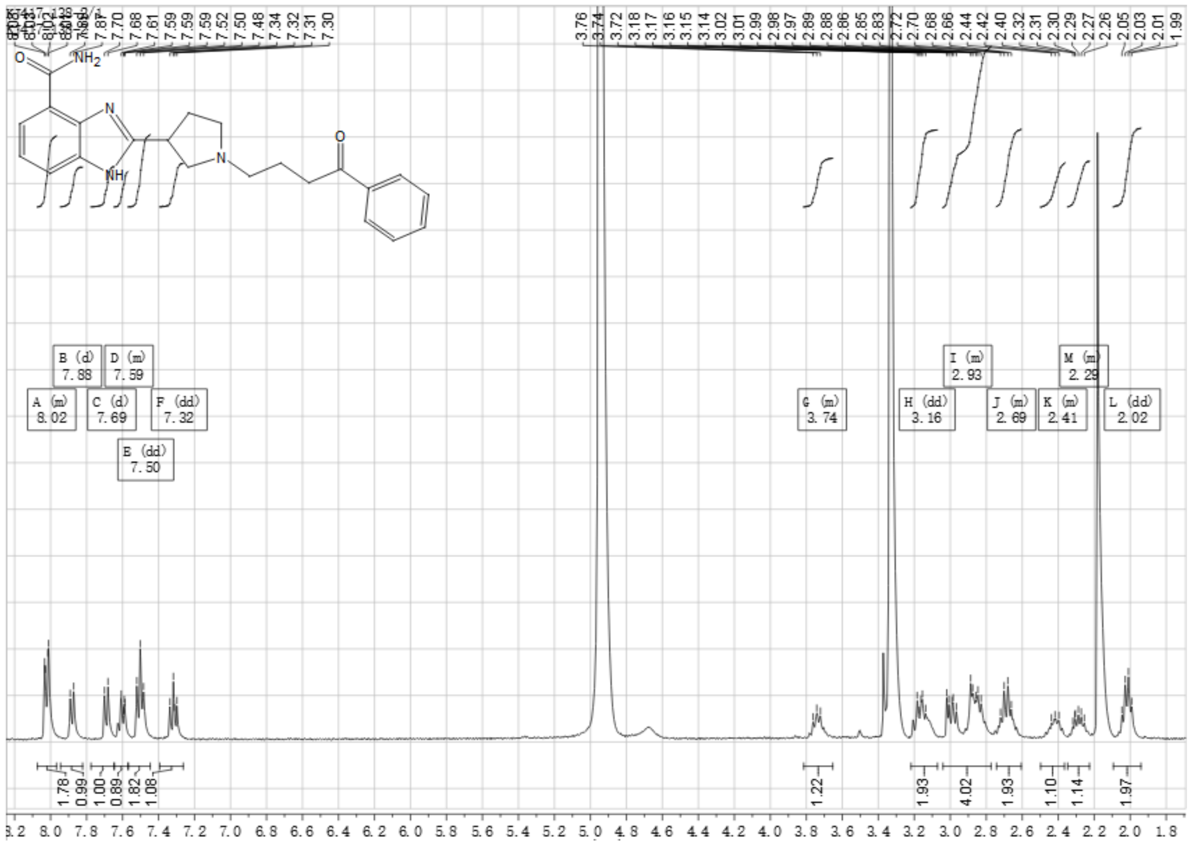

^{13}C NMR (101 MHz, MeOD) δ 158.48, 132.79, 128.30, 127.72, 122.16, 121.39, 58.39, 55.02, 53.28, 48.44, 48.23, 36.97, 29.68, 22.85.

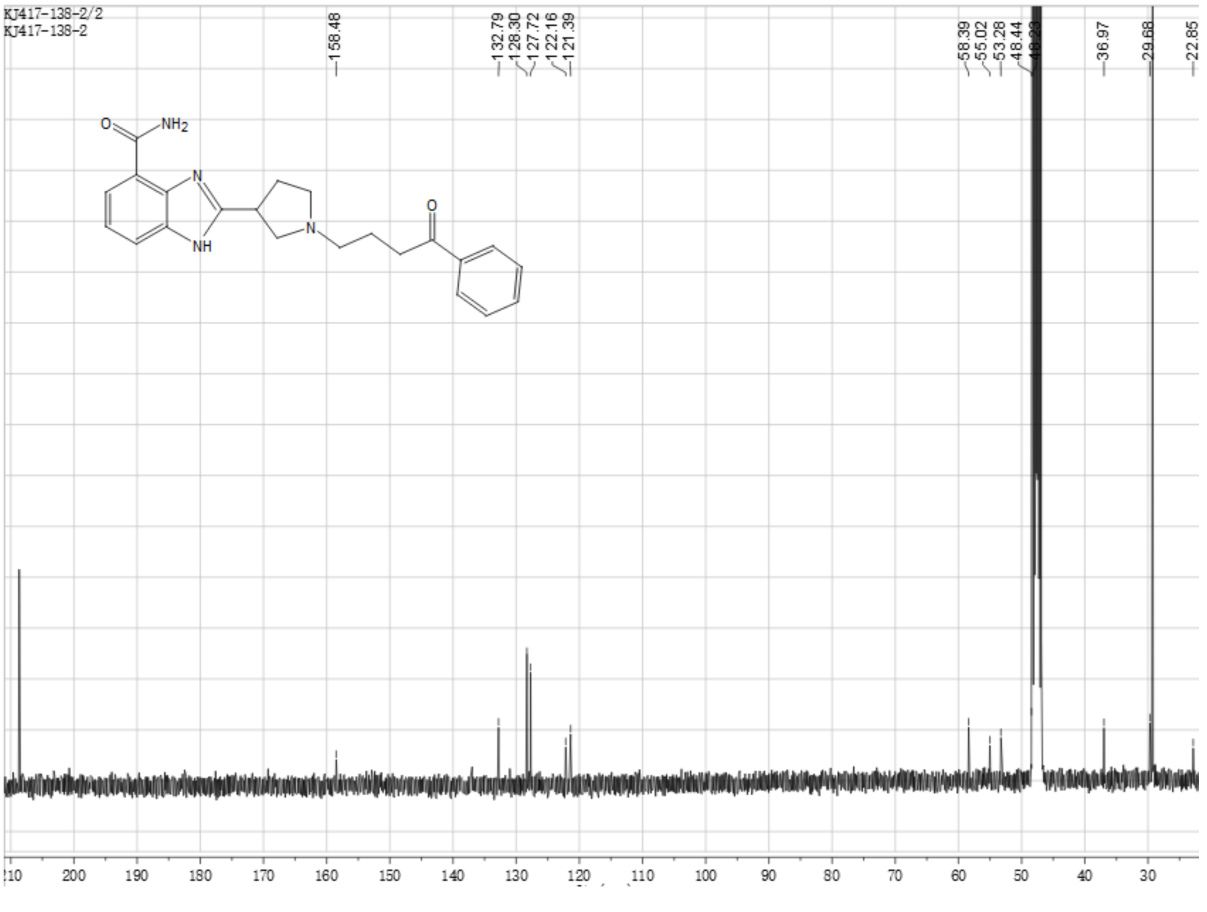

5cd

^{1}H NMR (400 MHz, DMSO-d_6) δ 12.71 (br, 1H), 9.30 (br, 1H), 8.05 – 7.93 (m, 2H), 7.79 (d, J = 7.6 Hz, 1H), 7.73 – 7.59 (m, 3H), 7.51 (dd, J = 7.7 Hz, 2H), 7.25 (dd, J = 7.8 Hz, 1H), 4.21 – 4.03 (m, 2H), 3.79 – 3.64 (m, 1H), 3.26 – 3.13 (m, 1H), 3.02 – 2.85 (m, 2H), 2.79 (td, J = 8.6, 6.0 Hz, 1H), 2.37 – 2.15 (m, 2H).

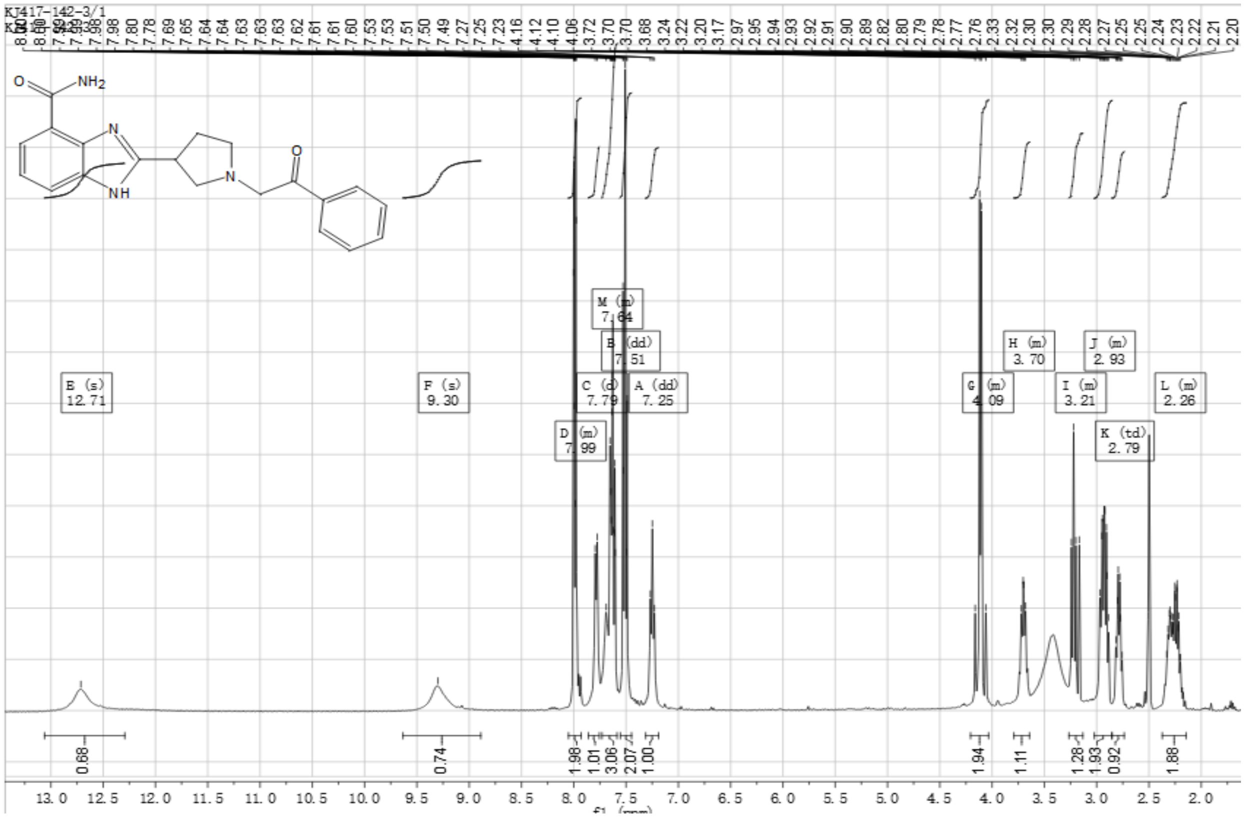

^{13}C NMR (101 MHz, DMSO) δ 197.41, 167.05, 158.73, 136.19, 133.68, 133.25, 129.71, 129.11, 128.43, 122.39, 121.74, 114.79, 61.70, 58.92, 53.65, 40.43, 37.39, 29.47.

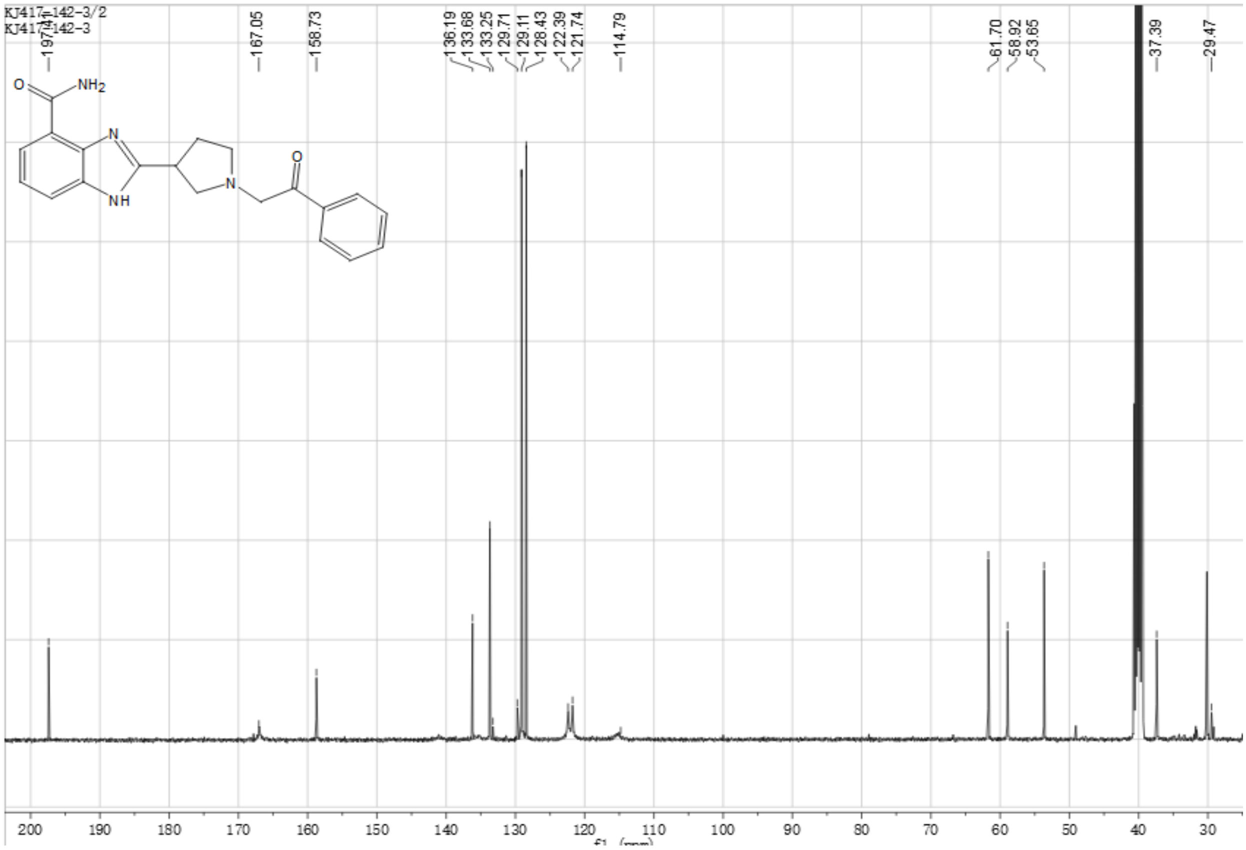

5ce

^{1}H NMR (400 MHz, Methanol-d_4) δ 7.88 – 7.80 (m, 3H), 7.78 – 7.73 (m, 2H), 7.66 (dd, J = 8.0, 1.1 Hz, 1H), 7.28 (t, J = 7.8 Hz, 1H), 3.80 (td, J = 6.9, 1.8 Hz, 2H), 3.65 (dd, J = 9.8, 7.6 Hz, 1H), 3.11 – 3.02 (m, 1H), 2.98 (dd, J = 9.5, 6.5 Hz, 1H), 2.78 (s, 2H), 2.70 – 2.60 (m, 2H), 2.34 (d, J = 10.0 Hz, 1H), 2.21 – 2.11 (m, 1H), 1.98 – 1.90 (m, 2H).

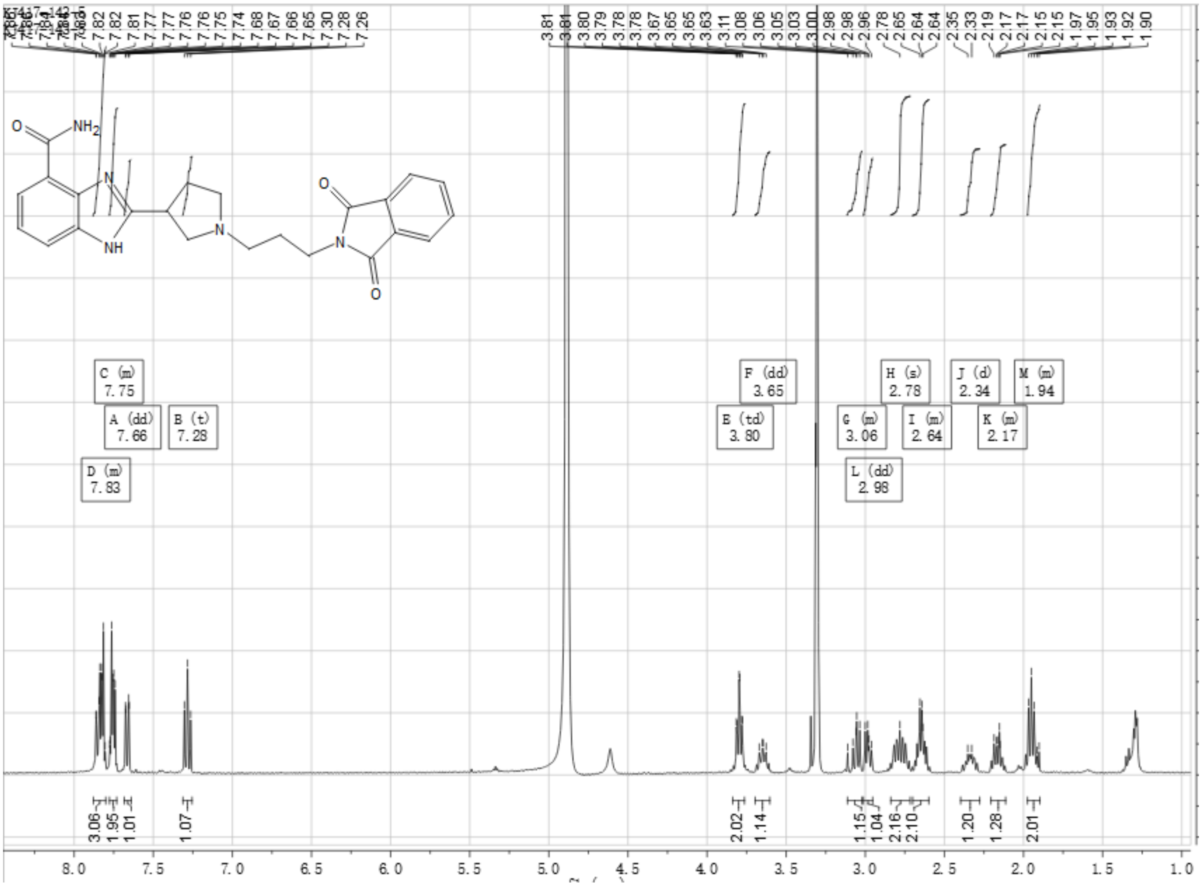

^{13}C NMR (101 MHz, MeOD) δ 169.26, 168.58, 158.51, 133.89, 132.00, 122.64, 122.19, 121.40, 58.28, 53.22, 53.03, 36.94, 35.83, 29.74, 26.83.

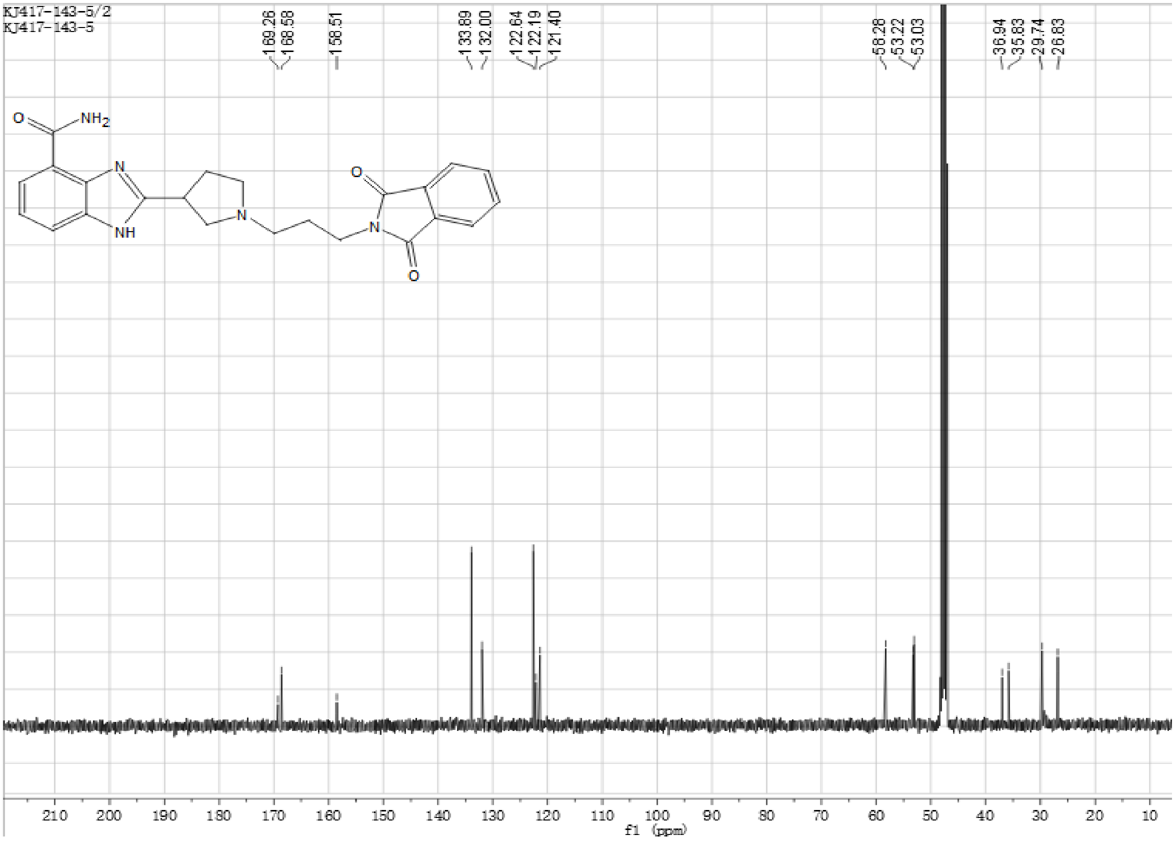

5cf

^{1}H NMR (400 MHz, Methanol-d_4) δ 7.87 (d, J = 7.6 Hz, 1H), 7.70 – 7.63 (m, 1H), 7.46 – 7.39 (m, 2H), 7.39 – 7.25 (m, 4H), 4.87 – 4.82 (m, 1H), 3.81 – 3.63 (m, 1H), 3.22 – 3.01 (m, 2.5H), 2.98 – 2.79 (m, 2.5H), 2.77 – 2.64 (m, 1H), 2.49 – 2.36 (m, 1H), 2.29 – 2.15 (m, 1H).

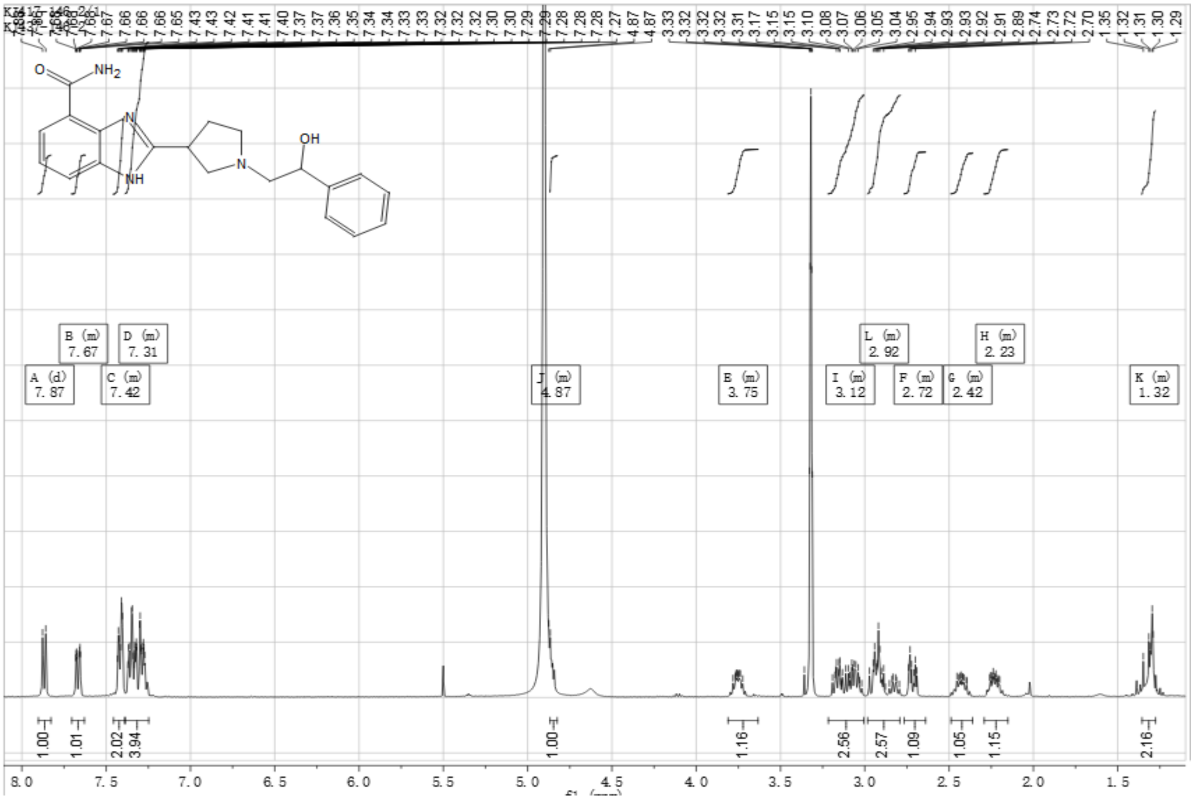

^{13}C NMR (101 MHz, MeOD) δ 169.29, 158.90, 143.36, 127.97, 127.16, 125.77, 122.17, 121.38, 100.00, 72.05, 63.33, 58.81, 53.76, 37.17, 29.35.

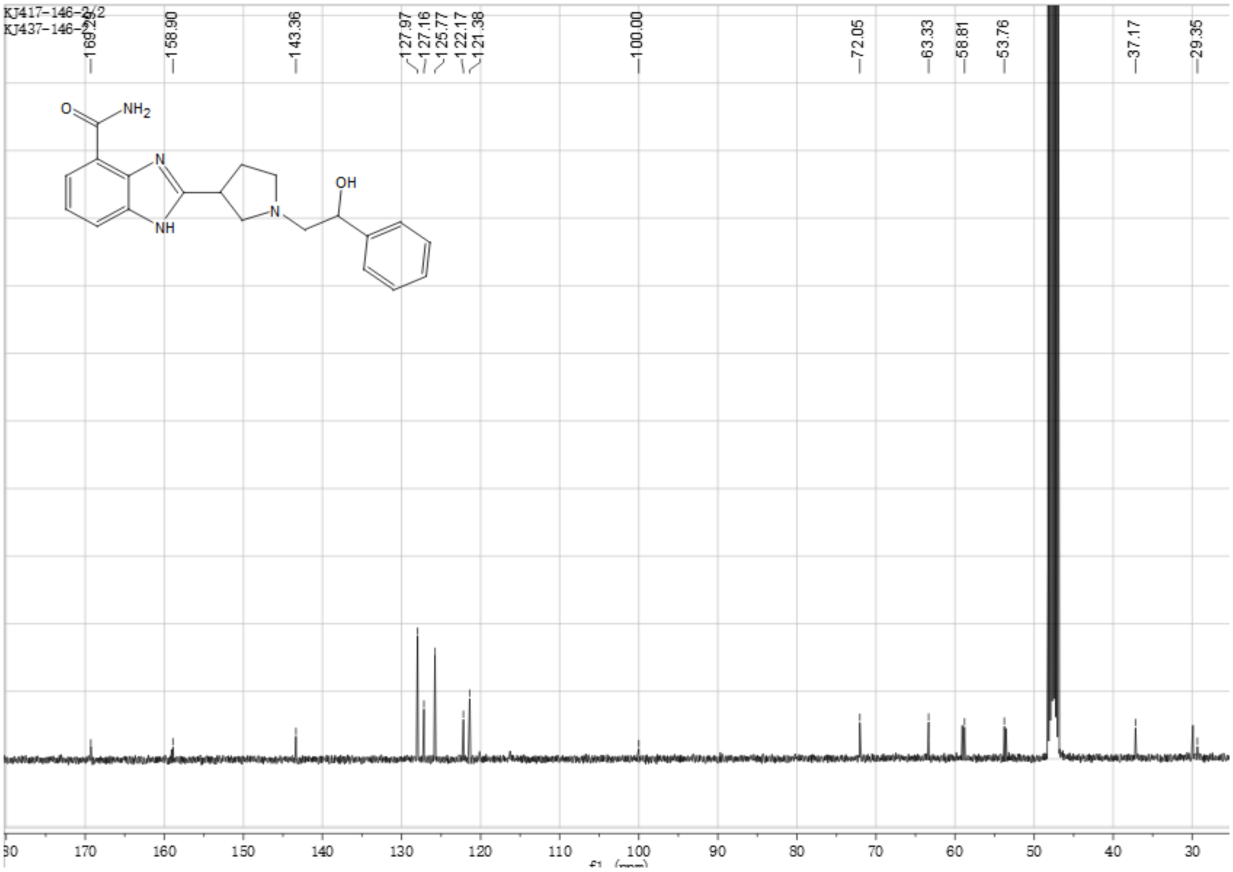

5cg

^{1}H NMR (400 MHz, Methanol-d_4) δ 7.86 (dd, J = 7.7, 1.0 Hz, 1H), 7.67 (dd, J = 8.0, 1.1 Hz, 1H), 7.41 – 7.21 (m, 6H), 4.81 – 4.76 (m, 1H), 3.87 – 3.74 (m, 1H), 3.25 – 3.13 (m, 1H), 3.05 – 2.98 (m, 2H), 2.96 – 2.86 (m, 1H), 2.85 – 2.75 (m, 1H), 2.54 – 2.43 (m, 1H), 2.39 – 2.27 (m, 1H), 2.12 – 1.94 (m, 3H).

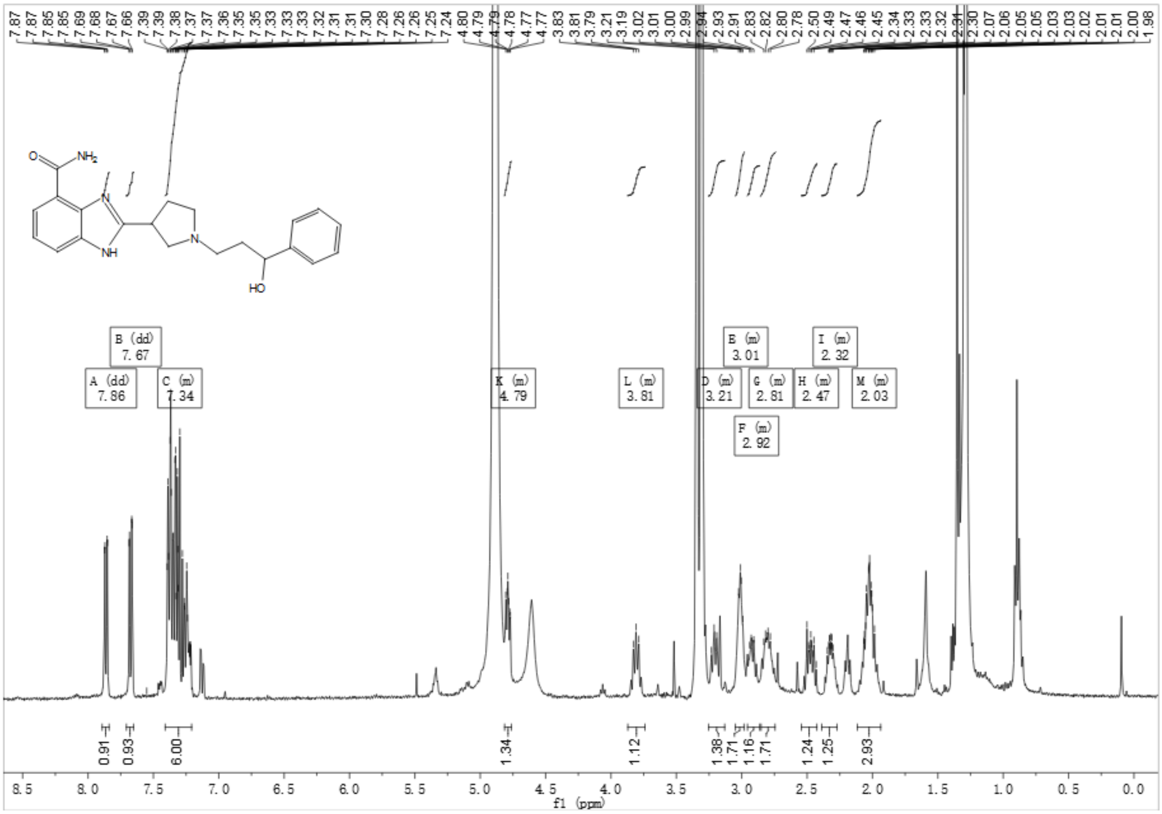

^{13}C NMR (101 MHz, MeOD) δ 158.42, 144.70, 127.88, 126.84, 125.50, 122.18, 121.36, 72.80, 58.52, 53.36, 52.76, 37.03, 36.96, 29.28.

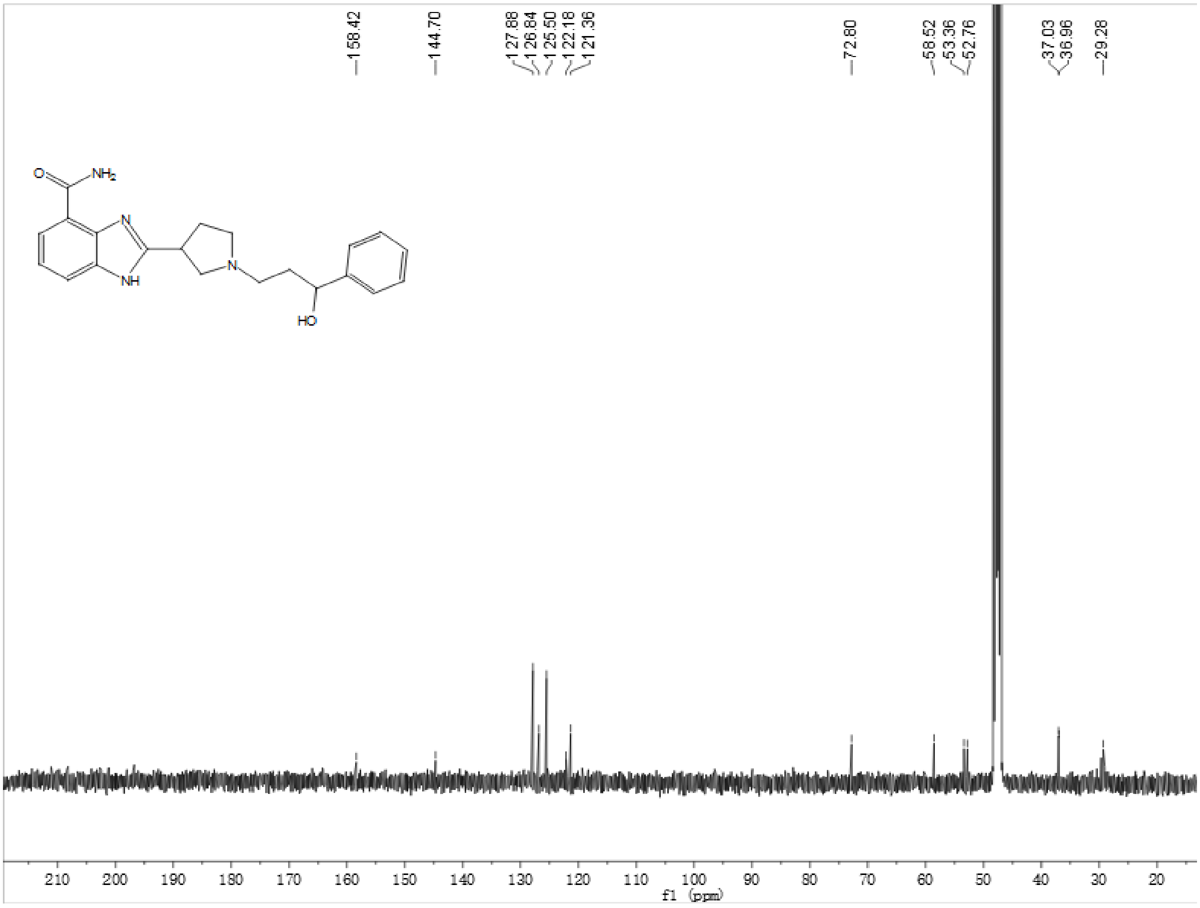

5ch

^{1}H NMR (400 MHz, DMSO-d_6) δ 9.19 (br, 1H), 7.76 (d, *J* = 7.5 Hz, 1H), 7.62 (d, *J* = 7.5 Hz, 2H), 7.23 (t, *J* = 7.8 Hz, 1H), 7.00 (t, *J* = 7.7 Hz, 2H), 6.47 (dd, *J* = 16.1, 7.8 Hz, 3H), 5.54 (br, 1H), 3.66 (dd, *J* = 9.6, 6.9 Hz, 2H), 3.02 (dd, *J* = 7.0 Hz, 2H), 2.86 – 2.76 (m, 1H), 2.70 (dd, *J* = 7.9 Hz, 2H), 2.57 (dd, *J* = 7.4 Hz, 2H), 2.33 – 2.13 (m, 2H), 1.72 (p, *J* = 7.0 Hz, 2H).

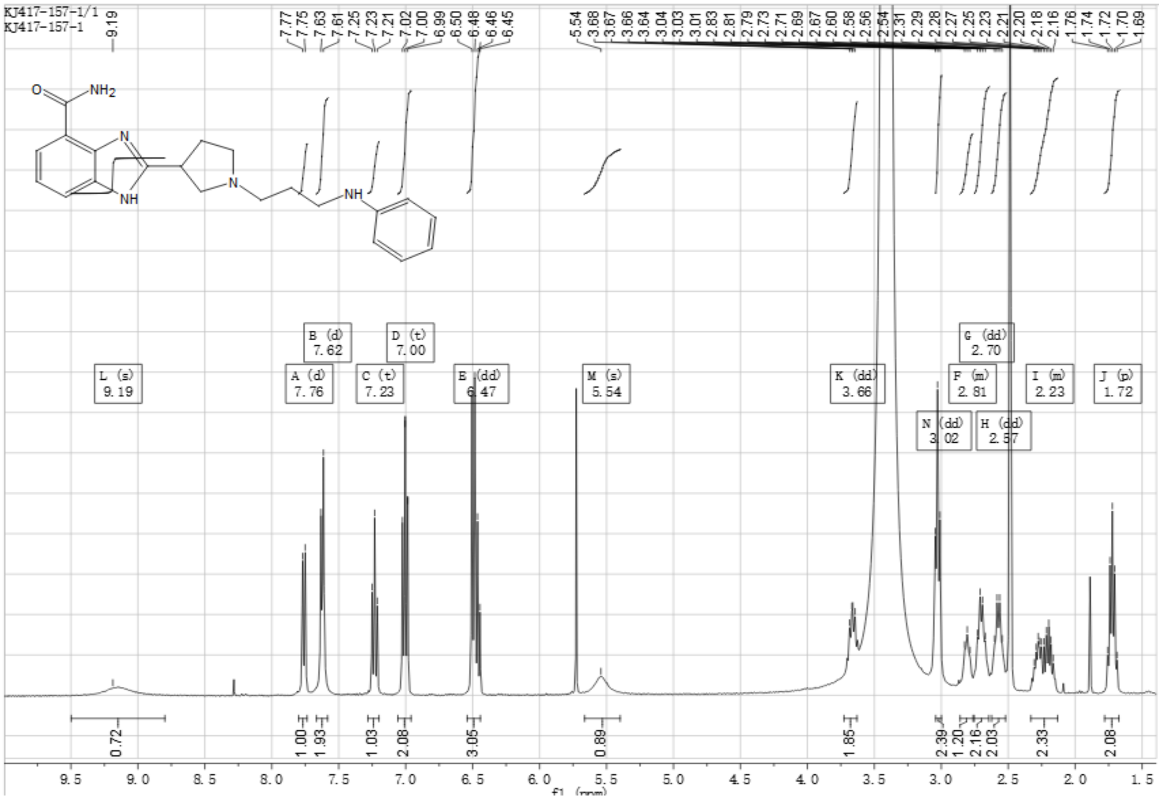

^{13}C NMR (101 MHz, DMSO) δ 167.12, 159.00, 149.47, 129.29, 122.36, 121.72, 115.87, 112.41, 59.22, 55.32, 53.82, 41.70, 37.19, 29.92, 28.18.

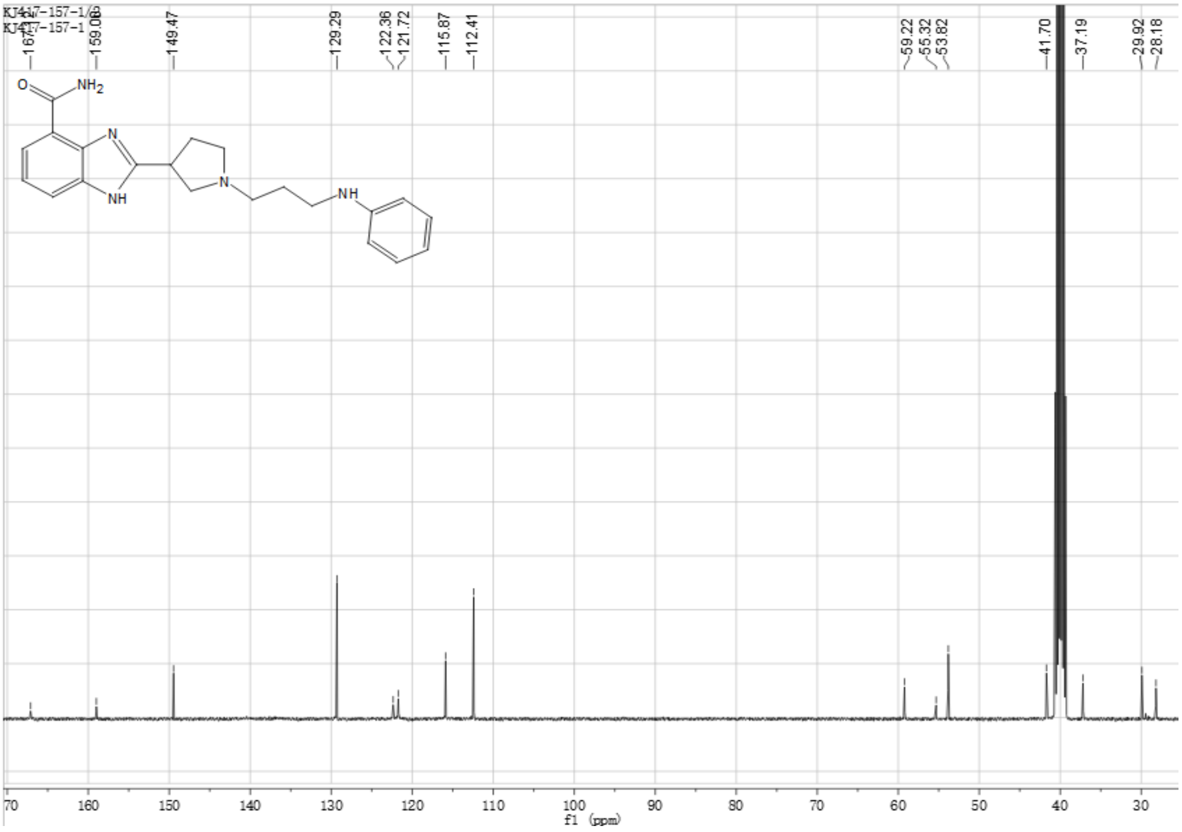

5ci

^{1}H NMR (400 MHz, Chloroform-*d*) δ 11.36 (br, 1H), 9.65 (br, 1H), 8.14 – 7.93 (m, 3H), 7.64 (d, *J* = 7.9 Hz, 1H), 7.30 (dd, *J* = 4.9, 3.8 Hz, 1H), 7.04 – 6.89 (m, 2H), 3.90 – 3.83 (m, 3H), 3.80 – 3.70 (m, 1H), 3.36 – 3.07 (m, 6H), 2.84 – 2.71 (m, 1H), 2.55 – 2.35 (m, 2H), 2.12 – 2.01 (m, 1H).

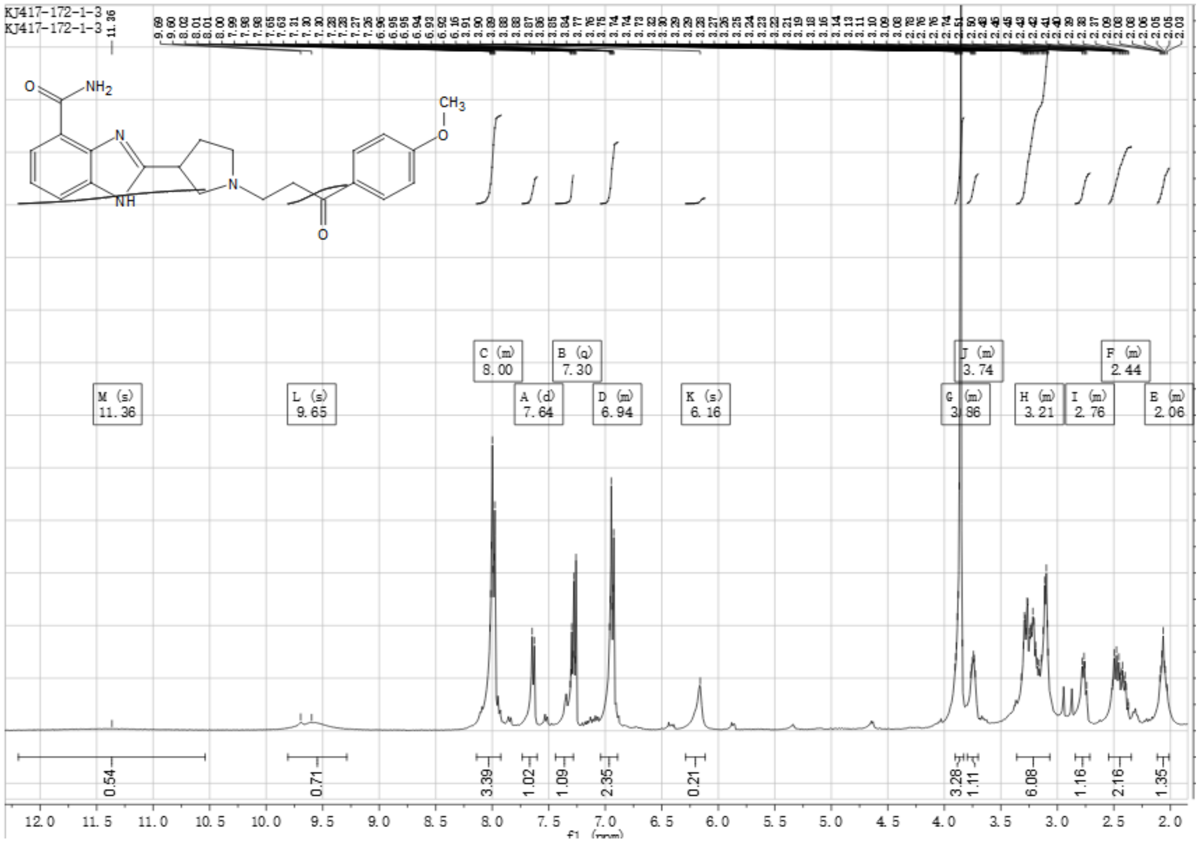

^{13}C NMR (101 MHz, MeOD) δ 197.88, 197.83, 169.21, 163.98, 158.17, 130.20, 129.50, 122.24, 121.47, 113.51, 58.43, 54.67, 53.43, 50.53, 36.97, 36.37, 29.79.

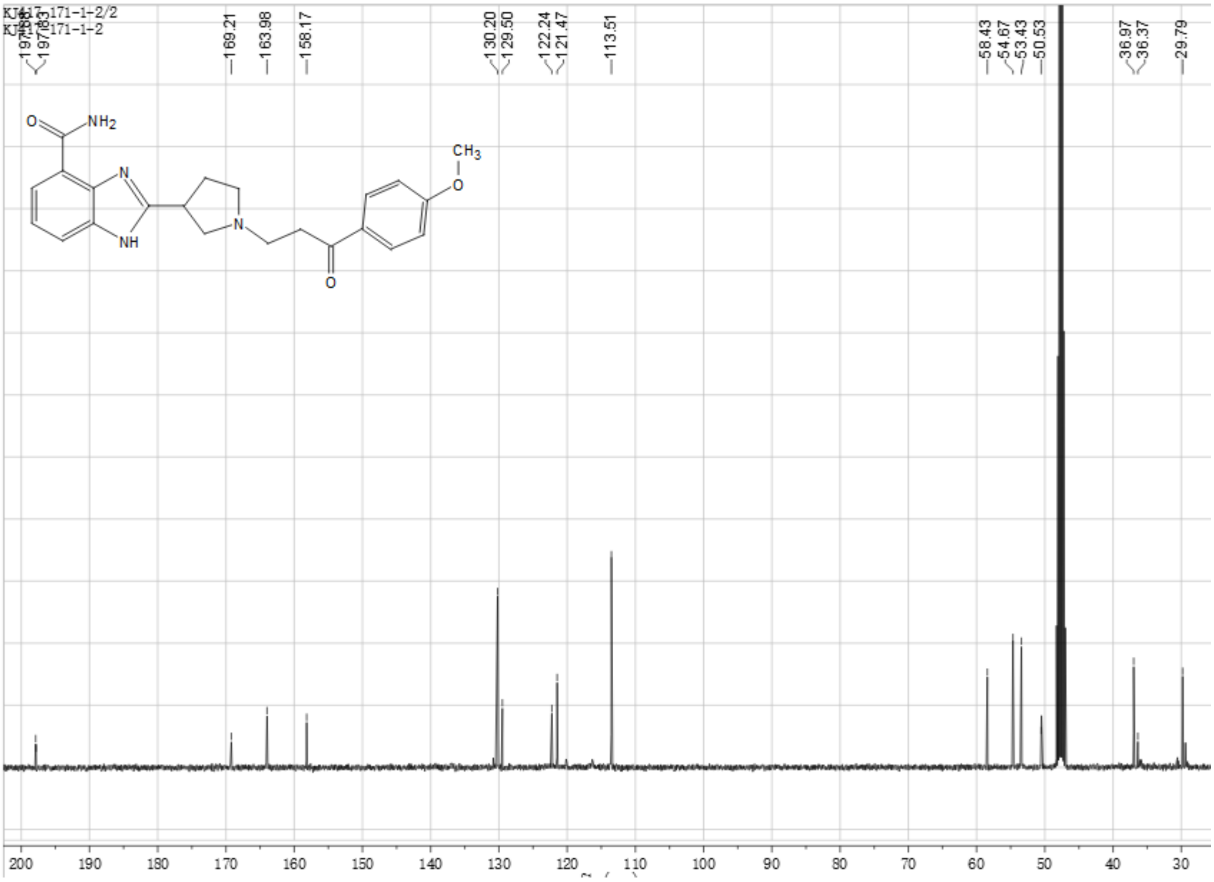

5cj

^{1}H NMR (400 MHz, Chloroform-*d*) δ 11.37 (br, 1H), 9.63 (br, 1H), 8.03 – 7.84 (m, 3H), 7.63 (d, *J* = 7.9 Hz, 1H), 7.46 – 7.40 (m, 2H), 7.30 – 7.23 (m, 1H), 3.77 – 3.68 (m, 1H), 3.36 – 3.00 (m, 6H), 2.77 (dd, *J* = 9.4, 6.7 Hz, 1H), 2.54 – 2.35 (m, 2H), 2.11 – 1.99 (m, 1H).

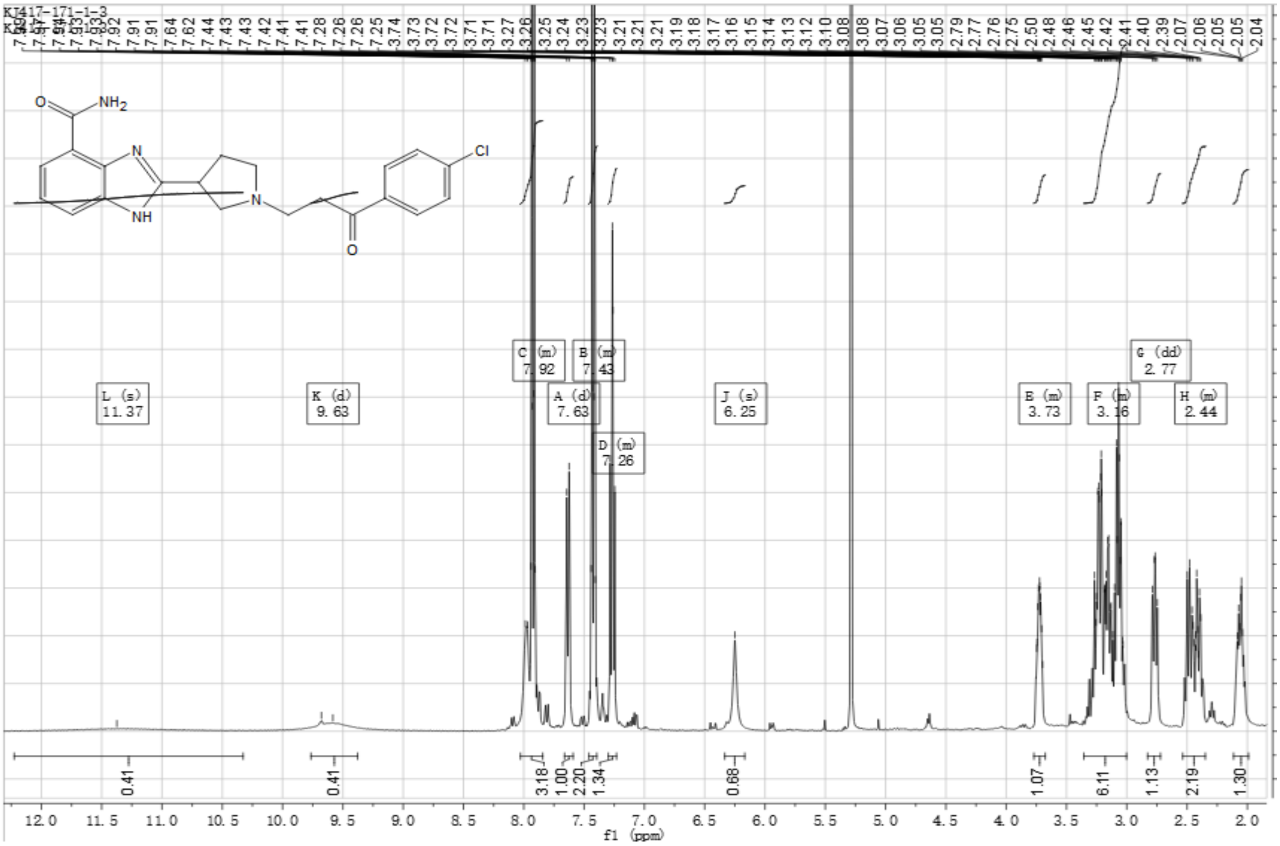

^{13}C NMR (101 MHz, MeOD) δ 197.96, 169.22, 158.18, 139.22, 135.19, 129.44, 128.58, 122.24, 121.46, 120.12, 116.39, 58.45, 53.41, 50.09, 37.02, 30.55, 29.35.

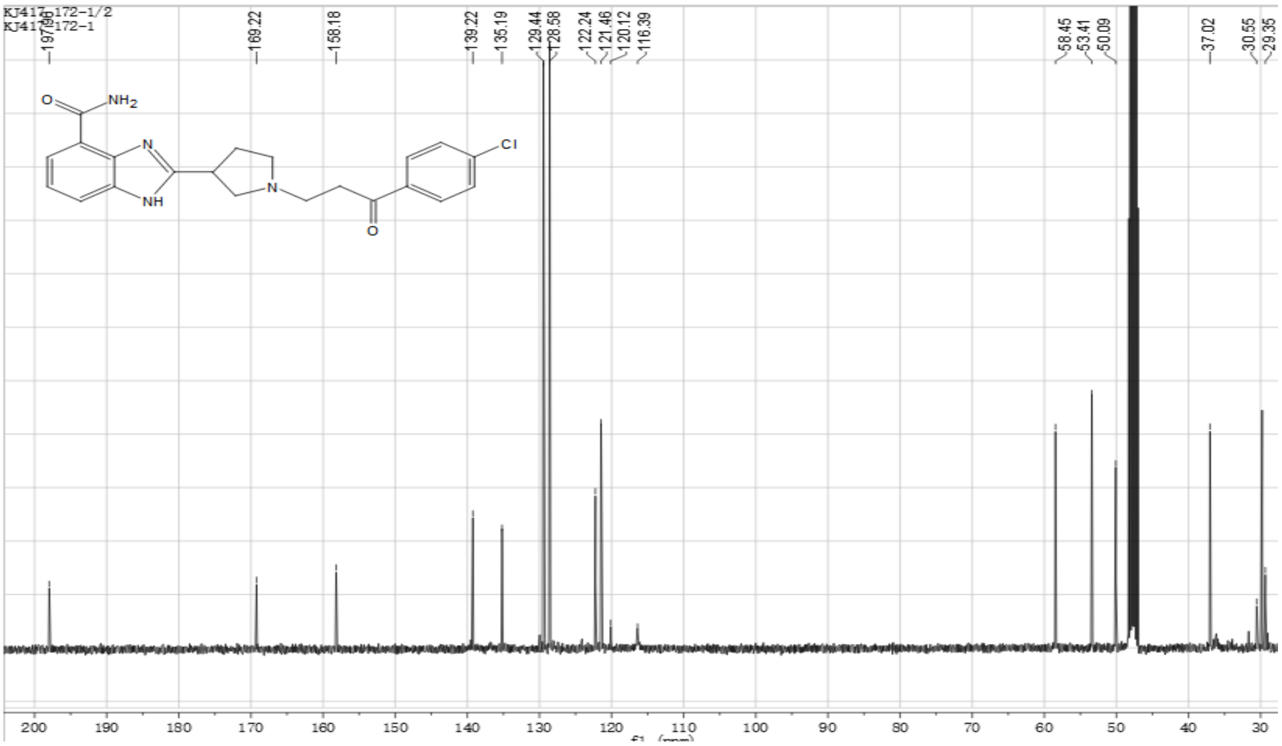

5ck

^{1}H NMR (400 MHz, Methanol-d_4) δ 7.88 – 7.78 (m, 3H), 7.78 – 7.72 (m, 2H), 7.62 (d, J = 8.0 Hz, 1H), 7.27 (dd, J = 7.8 Hz, 1H), 3.95 – 3.83 (m, 2H), 3.77 – 3.66 (m, 1H), 3.25 – 3.11 (m, 2H), 3.03 – 2.82 (m, 4H), 2.46 – 2.34 (m, 1H), 2.29 – 2.17 (m, 1H).

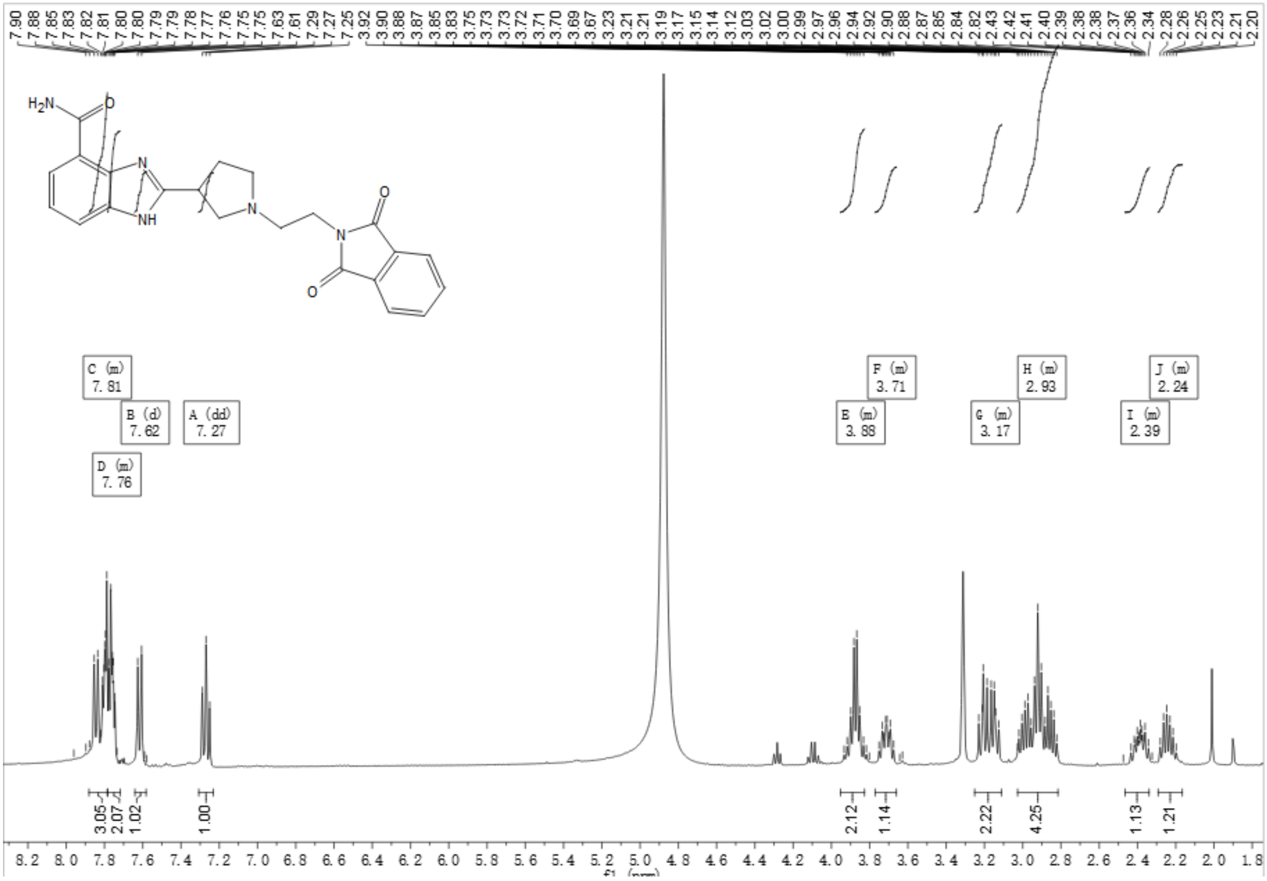

^{13}C NMR (101 MHz, MeOD) δ 169.19, 168.50, 158.24, 133.87, 132.03, 122.66, 122.21, 121.41, 58.20, 53.51, 53.23, 37.01, 36.13, 29.61.

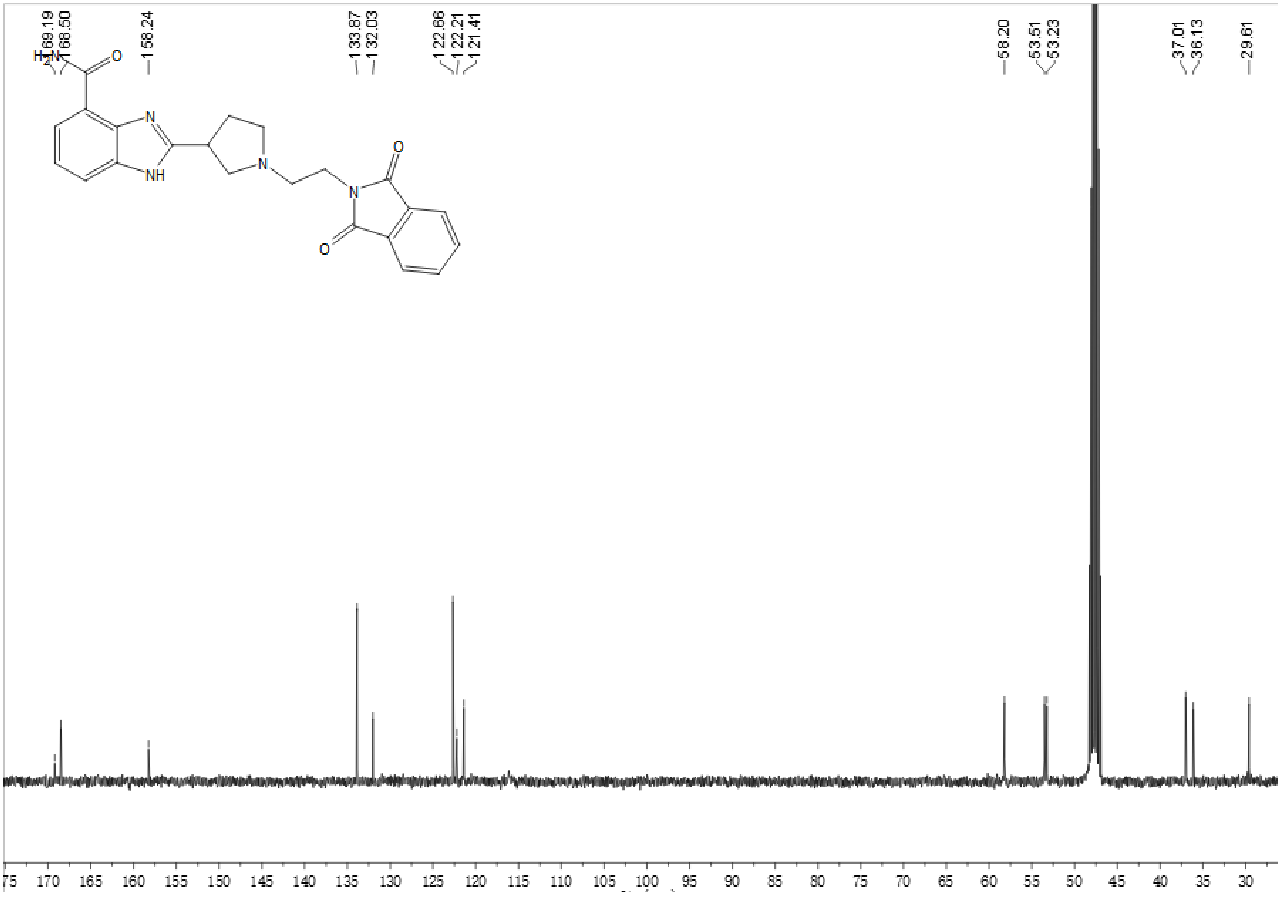

5cl

^{1}H NMR (400 MHz, Methanol-d_4) δ 7.86 (dd, J = 7.7, 1.1 Hz, 1H), 7.66 (dd, J = 8.0, 1.0 Hz, 1H), 7.28 (dd, J = 7.8 Hz, 1H), 3.80 – 3.65 (m, 1H), 3.11 (dd, J = 9.6, 8.0 Hz, 1H), 2.95 (dd, J = 9.6, 6.8 Hz, 1H), 2.88 – 2.71 (m, 4H), 2.71 – 2.53 (m, 2H), 2.46 – 2.35 (m, 1H), 2.33 – 2.18 (m, 1H), 1.75 (p, J = 7.3 Hz, 2H).

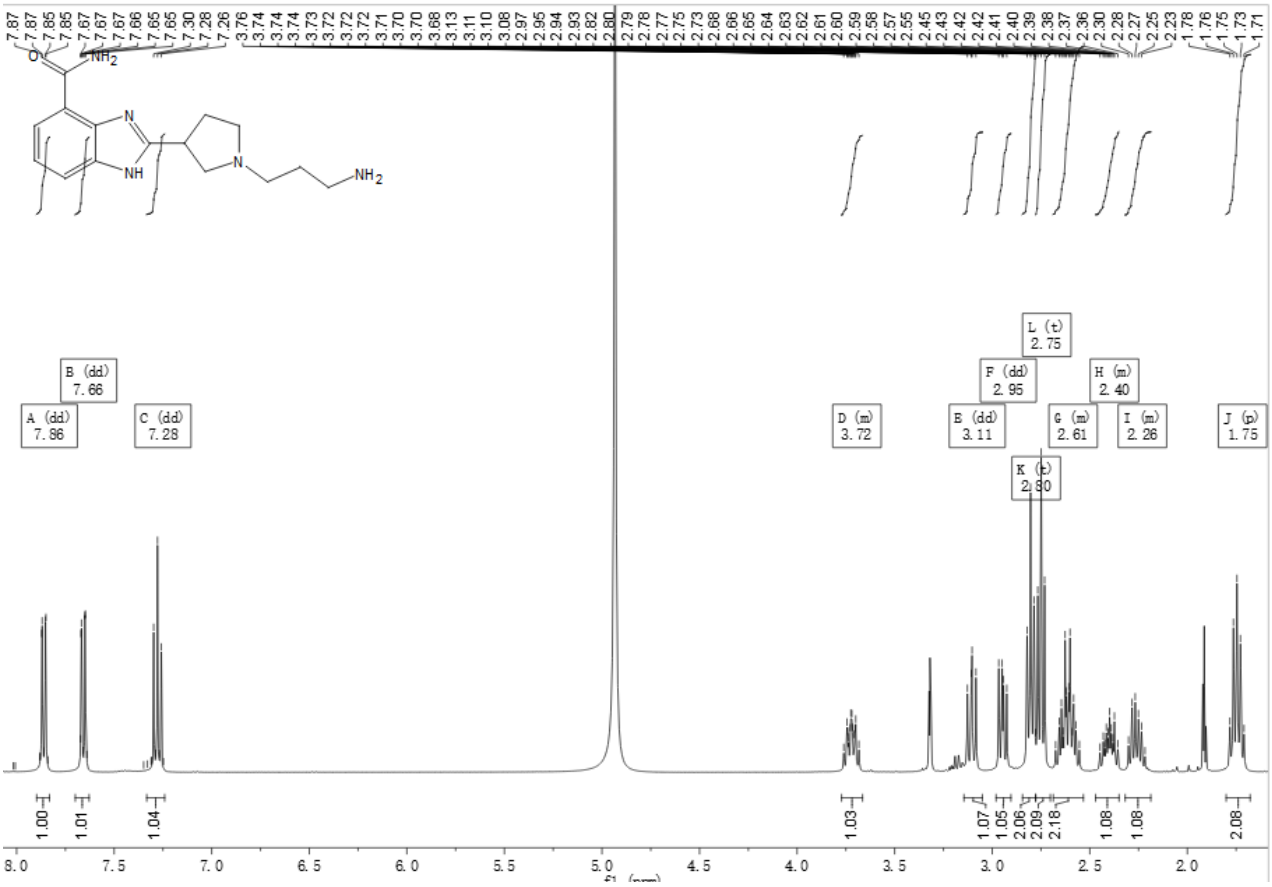

^{13}C NMR (101 MHz, MeOD) δ 169.29, 158.58, 122.16, 121.40, 120.00, 116.49, 58.56, 53.48, 53.44, 39.49, 36.93, 30.50, 29.71.

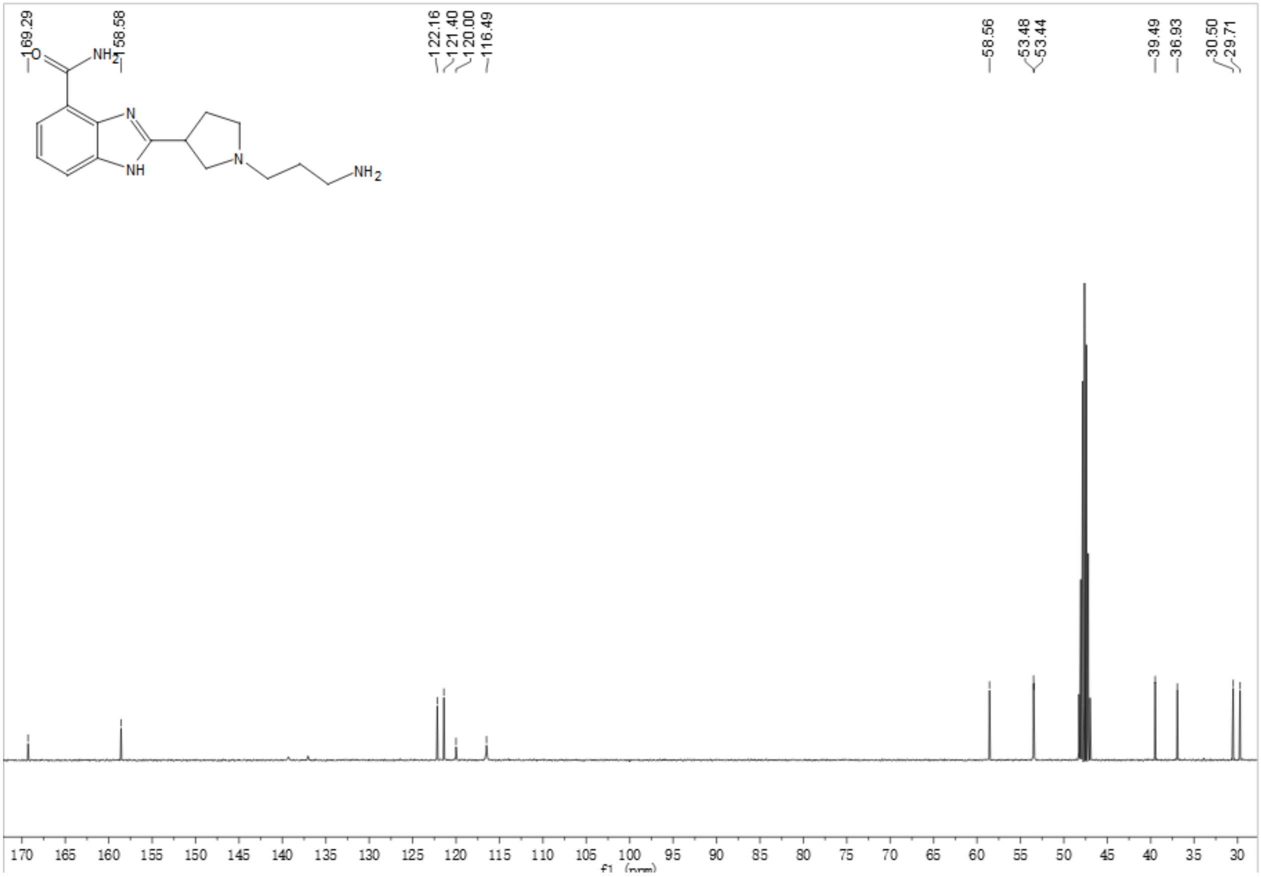

5cm

^{1}H NMR (400 MHz, Methanol-d_4) δ 7.88 – 7.84 (m, 1H), 7.68 – 7.64 (m, 1H), 7.28 (dd, J = 7.8 Hz, 1H), 3.78 – 3.67 (m, 1H), 3.08 (dd, J = 9.5, 7.9 Hz, 1H), 2.97 (dd, J = 9.5, 6.5 Hz, 1H), 2.89 – 2.75 (m, 4H), 2.72 – 2.61 (m, 2H), 2.47 – 2.34 (m, 1H), 2.29 – 2.18 (m, 1H).

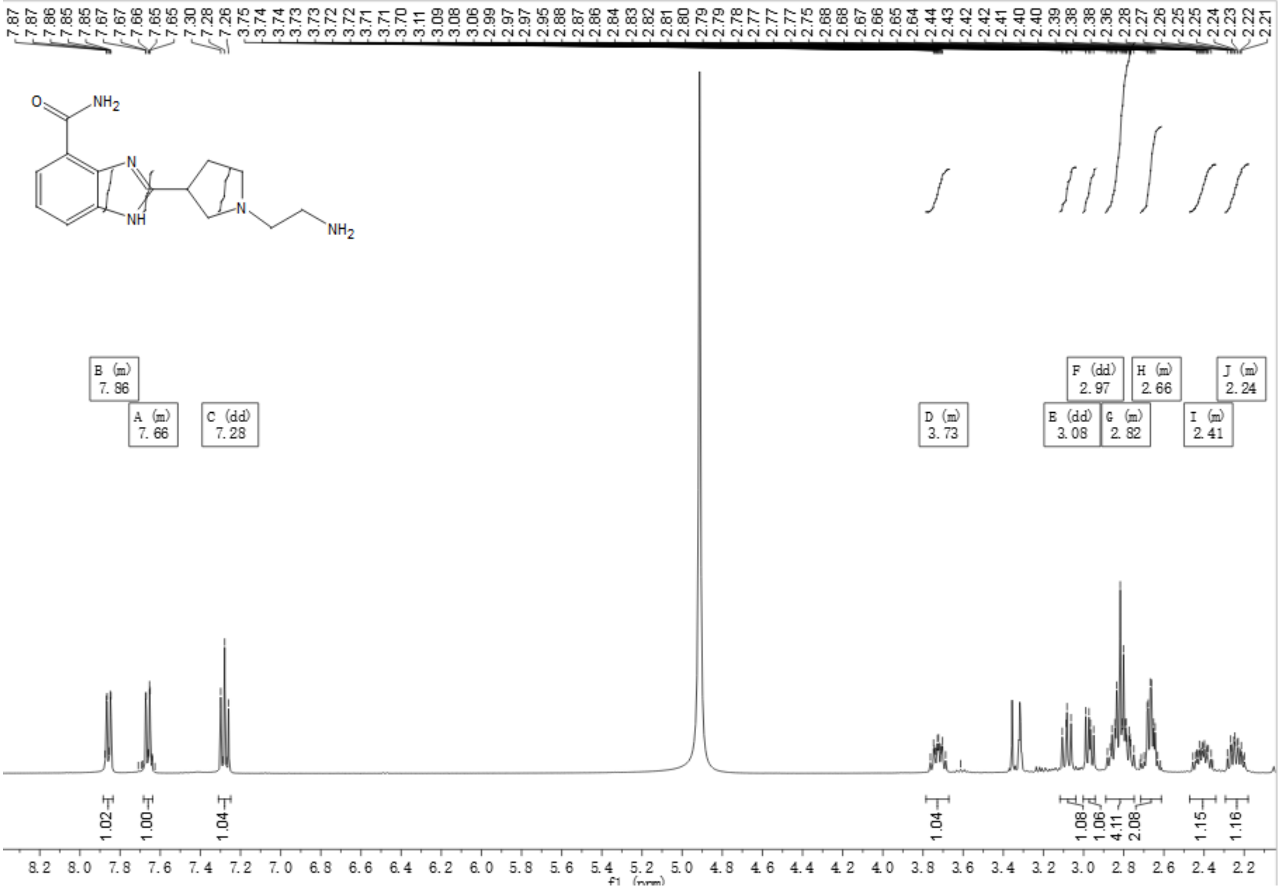

^{13}C NMR (101 MHz, MeOD) δ 169.30, 158.75, 121.39, 119.94, 116.55, 58.68, 57.63, 53.36, 39.51, 37.01, 29.83.

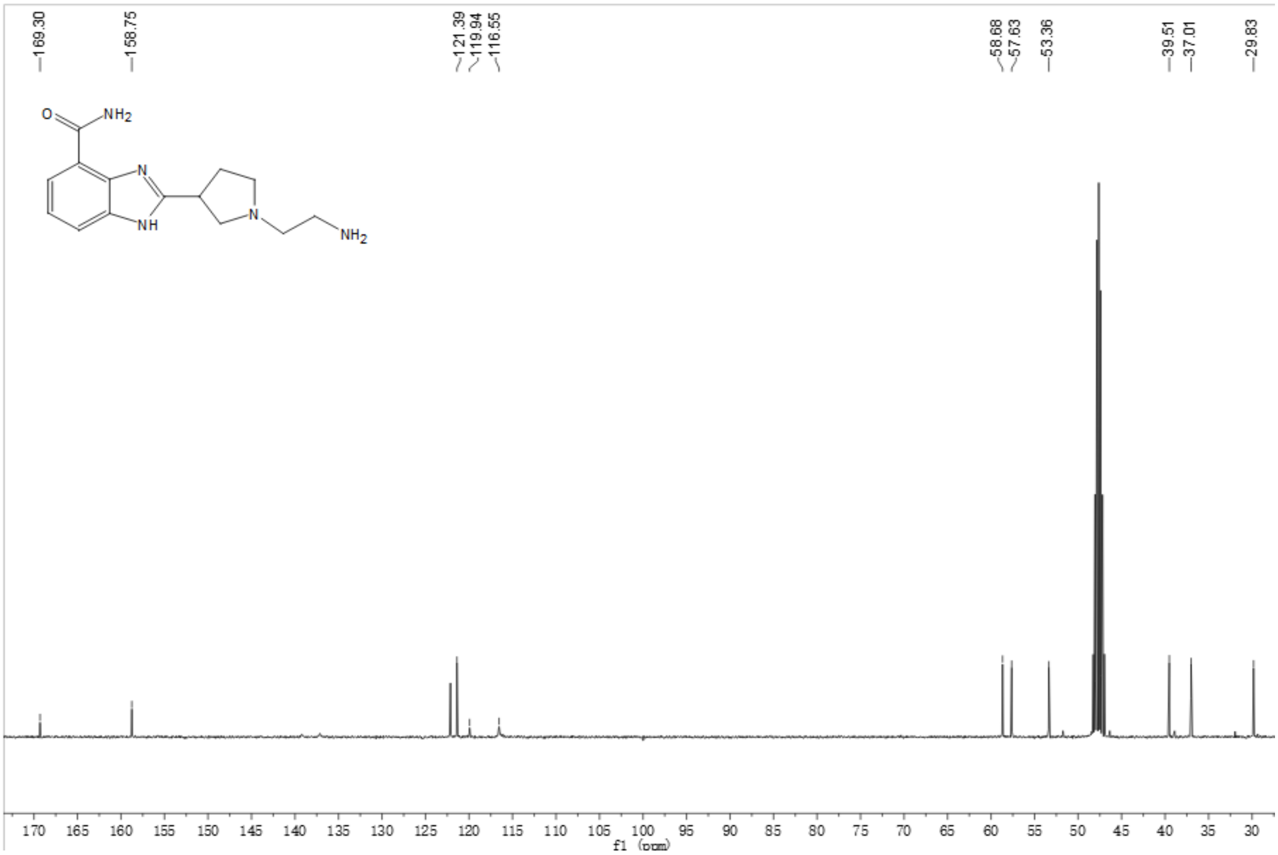

5cn

^{1}H NMR (400 MHz, Methanol-d_4) δ 7.87 (dd, J = 7.7, 1.0 Hz, 1H), 7.85 – 7.79 (m, 2H), 7.69 (dd, J = 8.0, 1.1 Hz, 1H), 7.56 – 7.49 (m, 1H), 7.47 – 7.41 (m, 2H), 7.31 (dd, J = 7.9 Hz, 1H), 3.99 – 3.88 (m, 1H), 3.60 – 3.48 (m, 4H), 3.30 – 3.17 (m, 2H), 3.11 – 3.01 (m, 2H), 2.65 – 2.52 (m, 1H), 2.44 – 2.32 (m, 1H), 2.09 – 1.97 (m, 2H).

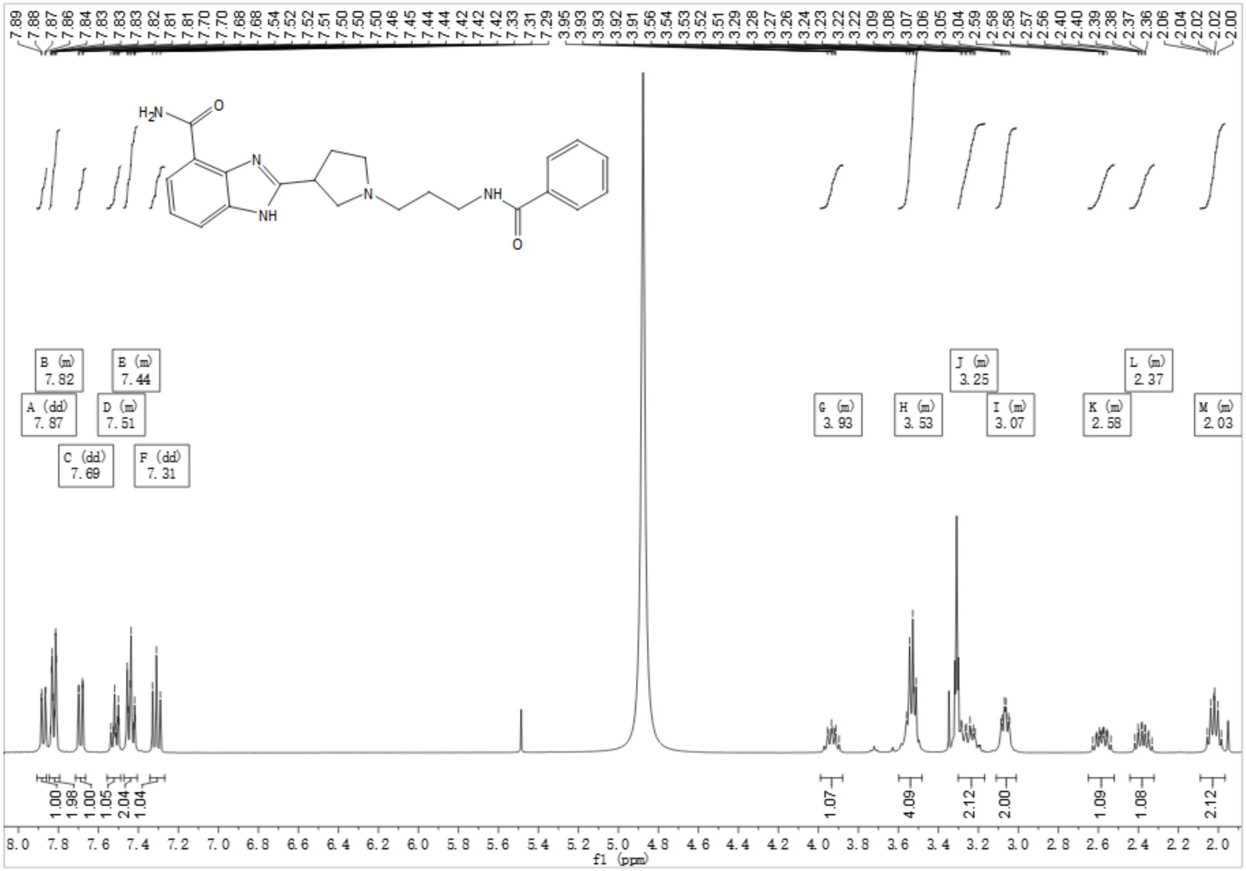

^{13}C NMR (101 MHz, MeOD) δ 169.27, 156.57, 133.92, 131.42, 128.20, 126.87, 122.48, 121.75, 57.66, 53.56, 53.23, 37.09, 36.61, 29.66, 26.99.

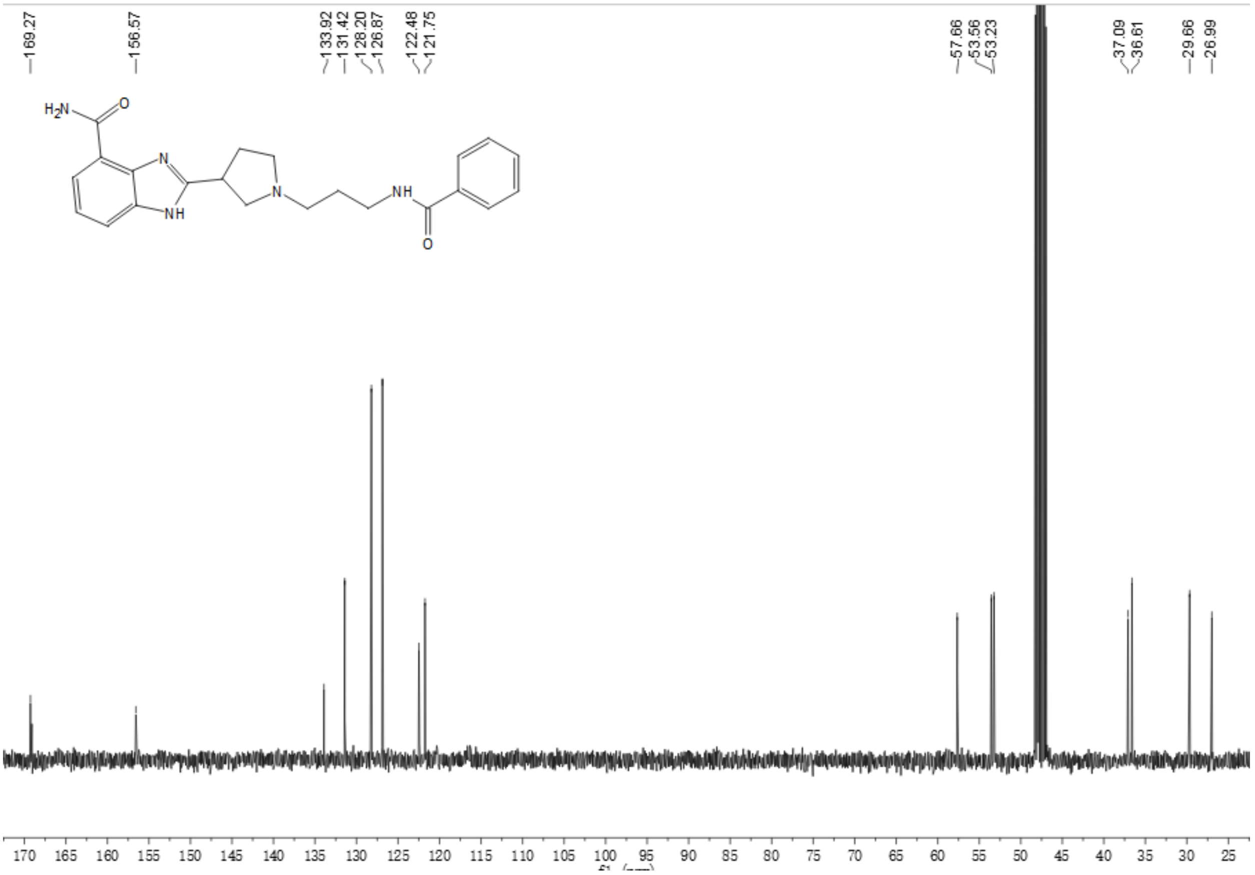

5co

^{1}H NMR (400 MHz, Methanol-d_4) δ 7.93 – 7.85 (m, 3H), 7.68 (d, *J* = 7.9 Hz, 1H), 7.55 (d, *J* = 7.3 Hz, 1H), 7.47 (dd, *J* = 7.6 Hz, 2H), 7.31 (dd, *J* = 7.8 Hz, 1H), 4.12 – 4.03 (m, 1H), 4.01 – 3.87 (m, 2H), 3.80 (t, *J* = 5.9 Hz, 2H), 3.71 – 3.57 (m, 2H), 3.56 – 3.47 (m, 2H), 2.74 – 2.62 (m, 1H), 2.52 – 2.40 (m, 1H).

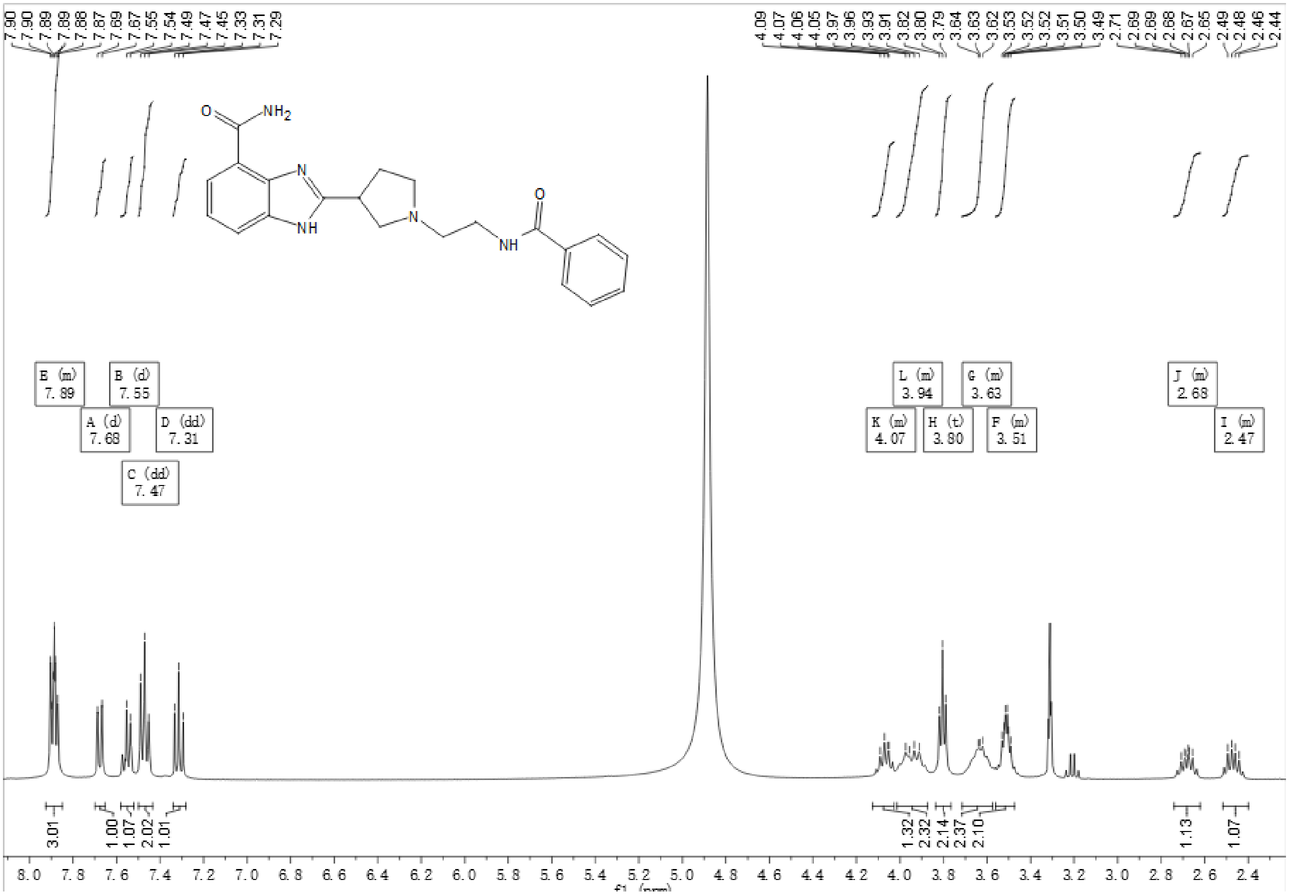

^{13}C NMR (101 MHz, MeOD) δ 169.64, 168.97, 155.15, 133.40, 131.73, 128.26, 127.12, 122.65, 121.95, 57.48, 55.25, 54.12, 36.51, 36.36, 29.49.

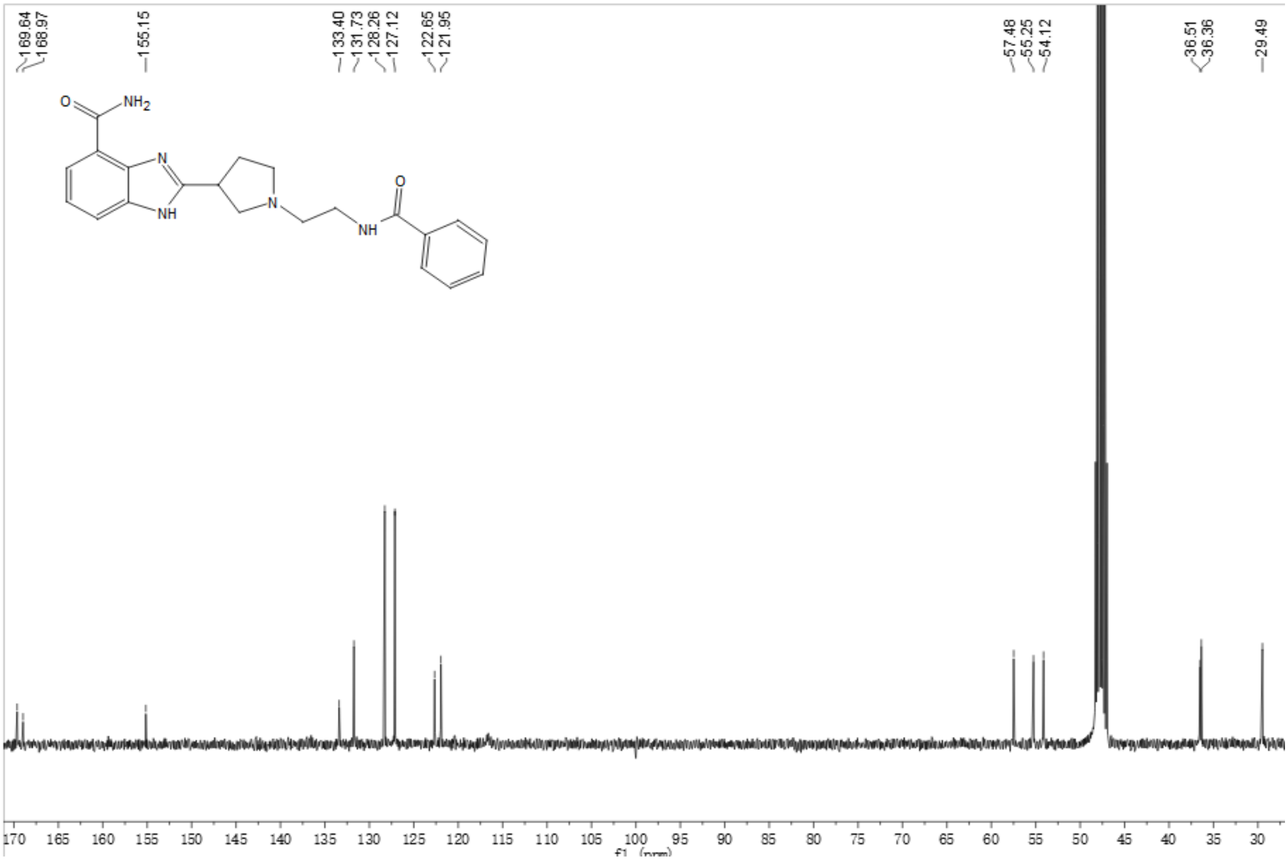

5cp

` ^{1}H NMR (400 MHz, DMSO-d_6) δ 9.10 (br, 1H), 8.05 – 7.94 (m, 2H), 7.80 (d, *J* = 7.5 Hz, 1H), 7.68 (td, *J* = 14.0, 5.3 Hz, 3H), 7.54 (t, *J* = 7.7 Hz, 2H), 7.26 (t, *J* = 7.7 Hz, 1H), 4.08 – 3.96 (m, 1H), 3.86 (s, 1H), 3.72 – 3.60 (m, 3H), 3.60 – 3.43 (m, 4H), 2.52 (s, 1H), 2.35 (s, 1H).

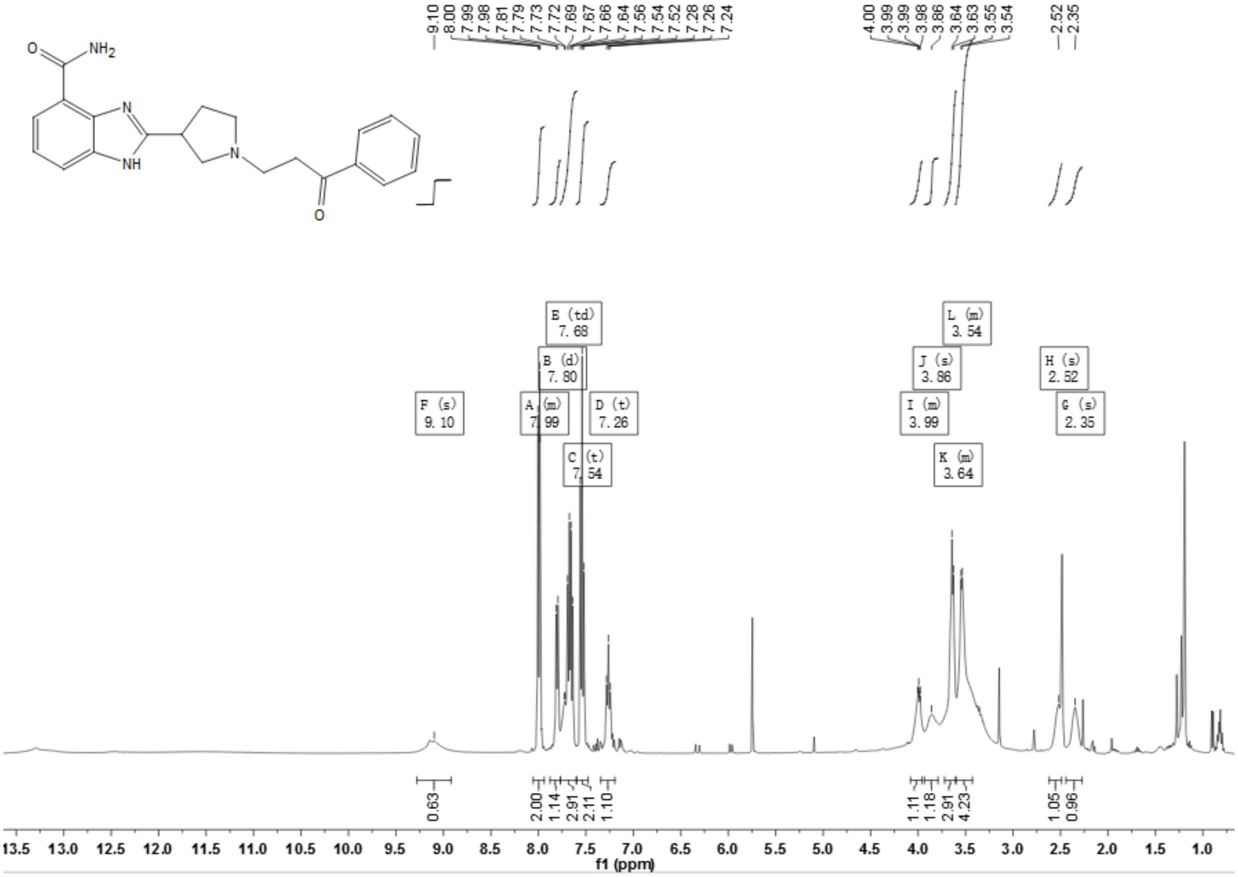

^{13}C NMR (101 MHz, DMSO) δ 197.43, 166.96, 155.69, 136.48, 134.16, 129.38, 129.31, 129.03, 128.70, 128.53, 122.69, 122.36, 122.13, 56.90, 53.56, 49.87, 36.59, 35.11, 29.83.

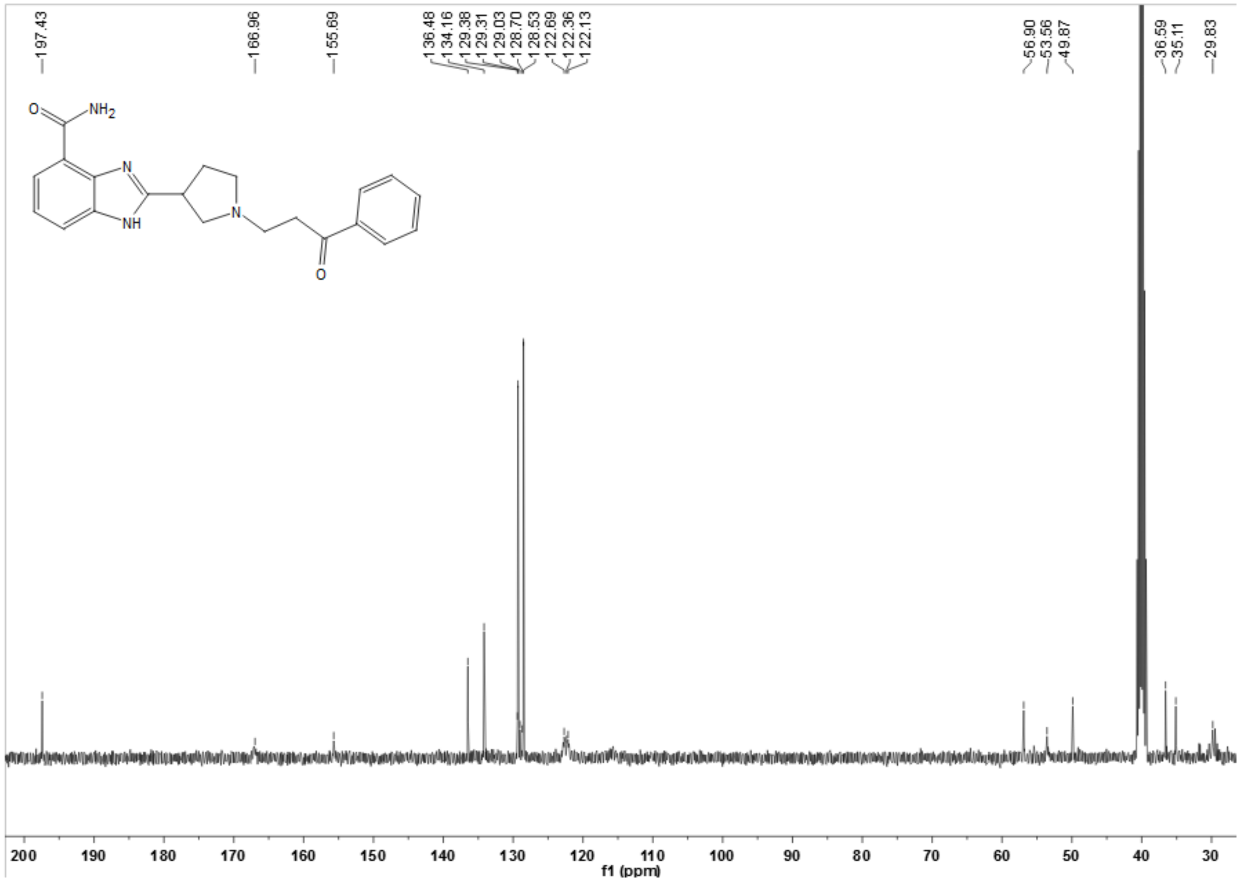

Silica gel

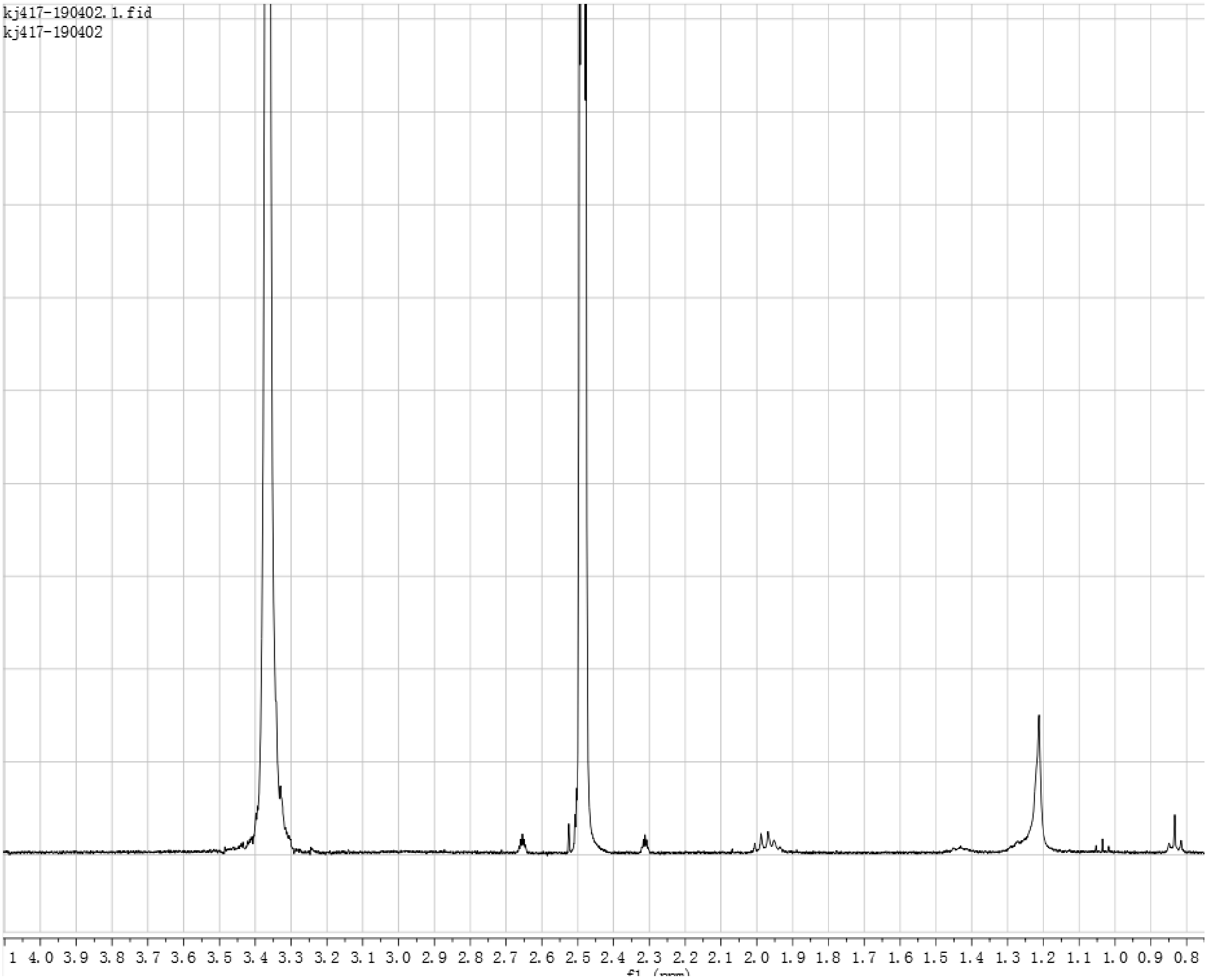

kj417-190402.1.fid
kj417-190402
1 4.0 3.9 3.8 3.7 3.6 3.5 3.4 3.3 3.2 3.1 3.0 2.9 2.8 2.7 2.6 2.5 2.4 2.3 2.2 2.1 2.0 1.9 1.8 1.7 1.6 1.5 1.4 1.3 1.2 1.1 1.0 0.9 0.8
